# PINK1-mediated phosphorylation of LETM1 regulates mitochondrial calcium transport and protects neurons against mitochondrial stress

**DOI:** 10.1038/s41467-017-01435-1

**Published:** 2017-11-09

**Authors:** En Huang, Dianbo Qu, Tianwen Huang, Nicoletta Rizzi, Wassamon Boonying, Dorothy Krolak, Paolo Ciana, John Woulfe, Christine Klein, Ruth S. Slack, Daniel Figeys, David S. Park

**Affiliations:** 1 University of Ottawa Brain and Mind Research Institute, Department of Cellular and Molecular Medicine, University of Ottawa, Ottawa, ON Canada K1H 8M5; 2 Center of Excellence on Neurodegenerative Diseases, Department of Pharmacological Sciences, University of Milan, Via Balzaretti 9, 20133 Milan, Italy; 3 Center of Excellence on Neurodegenerative Diseases, Department of Oncology and Hemato-Oncology, University of Milan, Via Balzaretti 9, 20133 Milan, Italy; 4 Centre for Cancer Therapeutics, Ottawa Hospital Research Institute and Department of Pathology and Laboratory Medicine, University of Ottawa, Ottawa, ON Canada K1H 8M5; 5 Institute of Neurogenetics, University of Lübeck, Ratzeburger Allee 160, 23538 Lübeck, Germany; 6 Department of Biochemistry, Microbiology and Immunology, Department of Chemistry and Biomolecular Sciences, and Ottawa Institute of Systems Biology, University of Ottawa, Ottawa, ON Canada K1H 8M5

## Abstract

Mutations in PTEN-induced kinase 1 (PINK1) result in a recessive familial form of Parkinson’s disease (PD). PINK1 loss is associated with mitochondrial Ca^2+^ mishandling, mitochondrial dysfunction, as well as increased neuronal vulnerability. Here we demonstrate that PINK1 directly interacts with and phosphorylates LETM1 at Thr192 in vitro. Phosphorylated LETM1 or the phospho-mimetic LETM1-T192E increase calcium release in artificial liposomes and facilitates calcium transport in intact mitochondria. Expression of LETM1-T192E but not LETM1-wild type (WT) rescues mitochondrial calcium mishandling in PINK1-deficient neurons. Expression of both LETM1-WT and LETM1-T192E protects neurons against MPP^+^–MPTP-induced neuronal death in PINK1 WT neurons, whereas only LETM1-T192E protects neurons under conditions of PINK1 loss. Our findings delineate a mechanism by which PINK1 regulates mitochondrial Ca^2+^ level through LETM1 and suggest a model by which PINK1 loss leads to deficient phosphorylation of LETM1 and impaired mitochondrial Ca^2+^ transport..

## Abstract (web summary)

Mutations in the mitochondrial kinase PINK1 result in familial Parkinson’s disease. Here the authors show that LETM1, a mitochondrial inner membrane protein, is a substrate of PINK1 that regulates Ca^2+^ handling in mitochondria in response to mitochondrial toxins.

## Introduction

PTEN-induced kinase 1 (PINK1) is a mitochondria targeted serine/threonine kinase. Mutations in PINK1 can cause recessive familial Parkinson’s disease (PD)^1, 2^. Under basal conditions, PINK1 is imported into the inner mitochondrial membrane, where it is processed by mitochondrial proteases^3–5^. However, upon mitochondrial membrane depolarization or damage, PINK1 accumulates on the outer membrane of mitochondria, where it recruits Parkin to trigger a mitophagic pathway of quality control^6^. Whether PINK1 exerts a biological function endogenously at the inner mitochondrial membrane is unknown. Previous evidence demonstrates that PINK1 loss results in mitochondrial dysfunction, reduced complex I activity, increased oxidative damage^7, 8^, mitochondrial Ca^2+^ ([Ca^2+^]_m_) mishandling and accumulation^9–11^, and increase in mPTP opening^11, 12^. One interpretation is that these changes may render dopaminergic (DA) neurons more vulnerable to stress and thereby contribute to the pathogenesis of PD^13, 14^.

To better define the mechanism(s) by which PINK1 may function at the mitochondria, we performed a mass spectrometry-based interactomics screen for potential PINK1-interacting proteins^15, 16^. One identified target is the leucine zipper-EF-hand containing transmembrane protein 1(LETM1), which was recently proposed as a Ca^2+^/H^+^ antiporter by a genome-wide RNAi screen^17^. LETM1 is a mitochondrial inner membrane protein^18^ and several reports suggest that it mediates mitochondrial Ca^2+^ uptake and extrusion in a gradient-dependent manner^17, 19, 20^. In this regard, knockdown of LETM1 compromises the rate of mitochondrial Ca^2+^ uptake and extrusion, leading to an alteration of mitochondrial bioenergetics, metabolic signaling, and sensitization to cell death^19–21^. However, others have suggested that LETM1 plays an essential role in mitochondrial K^+^ homeostasis by mediating the mitochondrial K^+^/H^+^ exchange^22^.

Our study indicates that LETM1 is a substrate of PINK1 and that LETM1 plays a critical role in protecting neurons from exogenous stress. Importantly, we also provide evidence that PINK1-mediated phosphorylation of LETM1 is crucial for its reported capacity for mitochondrial Ca^2+^ regulation and neuronal survival effects. Taken together, our data provide a compelling model by which PINK1 at the inner mitochondrial membrane performs a critical function of regulating LETM1 function.

## Results

### PINK1 phosphorylates LETM1 at Thr192

**Fig. 1:**
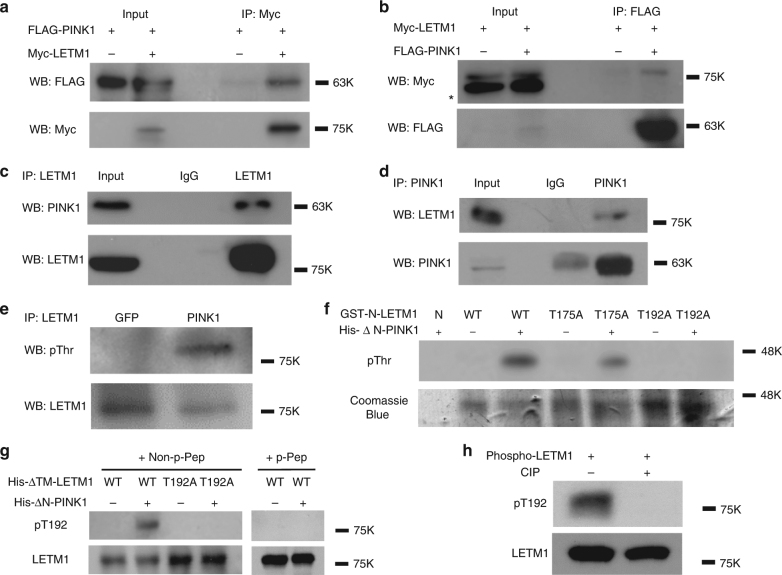
PINK1 interacts with and phosphorylates LETM1. **a** Expressed PINK1 interacts with LETM1. HEK293 cells were co-transfected with p3xFLAG-CMV-PINK1 and pCMV-3Tag-2a LETM1 (Myc-tag) for 1 day. Myc-LETM1 was IPed from cell lysate with anti-Myc antibody and probed with anti-FLAG antibody by WB. The membrane was reprobed with anti-Myc antibody. **b** Inversely to **a**, FLAG-PINK1 protein was IPed with anti-FLAG antibody and probed with anti-Myc and Anti-FLAG antibodies. * is non-special band. **c** Endogenous PINK1 interacts with LETM1 in human post-mortem brain tissue. Brain lysate was IPed with control IgG or anti-LETM1. The precipitated proteins were probed with anti-PINK1 and reprobed with anti-LETM1 antibodies by WB. **d** Inversely to **c** IP was performed with control IgG or anti-PINK1, probed with anti-LETM1 and reprobed with anti-PINK1 antibody by WB. **e** PINK1 phosphorylates LETM1 at threonine residue(s) in vivo. HEK293 cells were transfected with GFP or pAdtrack-PINK1 for 1 day. LETM1 was IPed with anti-LETM1 antibody, probed with anti-phospho-Thr and reprobed with anti-LETM1 antibody by WB. **f** PINK1 phosphorylates LETM1 at Thr192 in vitro. Purified bacterial GST-N-LETM1 variants (resides 1–204) were subjected to an in vitro kinase assay with bacterial His-∆N-PINK1 (residues 1–111 deleted) and probed with phospho-Thr antibody. Coomassie blue staining was performed as loading control for GST-N-LETM1. N: nil. **g** Bacterial His-∆N-LETM1 variants were subjected to in vitro kinase assay with His-∆N-PINK1 and probed for phospho-LETM1 utilizing the Thr192 (pT192) phospho-antibody. Samples were reprobed with anti-LETM1 for loading. **h** His-∆N-LETM1 was incubated with His-∆N-PINK1 in an in vitro kinase reaction to generate phospho-LETM1. Phospho-LETM1 was then treated without or with CIP for 2 h then probed with pT192 and reprobed with LETM1 antibody. All above experiments were replicated three times, respectively

We initially identified LETM1 as one of the PINK1-interacting candidates through an unbiased interaction screen. Briefly, PINK1-FLAG was expressed in HEK293 cells and immunoprecipitated. Interacting proteins were determined by mass spectrometry-based interactomics as previously reported^15, 16^. Analysis of the data set suggested LETM1 as one of the PINK1-interacting candidates with a Mascot score of 85. To validate this initial finding, we performed co-immunoprecipitation (Co-IP) analyses with expression of FLAG-tagged PINK1 and Myc-tagged LETM1 in HEK293 Cells (ATCC). As shown in Fig. 1a, Co-IP for Myc-LETM1 with anti-Myc antibody followed by Western blot (WB) analyses for FLAG-PINK1 with anti-FLAG antibody demonstrated a specific interaction. The reverse Co-IP also supported the interaction (Fig. 1b). We further confirmed the interaction of endogenous PINK1 with LETM1 from human post-mortem brain (Fig. 1c, d) and human SH-SY5Y neuroblastoma cells (Supplementary Fig. 1a, b). We next examined whether LETM1 may be a kinase substrate of PINK1. To test this, endogenous LETM1 from HEK293 cells expressing GFP or PINK1 was IPed with an anti-LETM1 antibody and probed with phospho-Thr and phospho-Ser antibodies by WB analyses. The results suggested that LETM1 was phosphorylated at threonine residues (Fig. 1e) but not serine (Supplementary Fig. 1c) in cells expressing PINK1 but not GFP. PINK1 distributes in the outer membrane, inner membrane, and intermembrane space of mitochondria but not in the matrix of mitochondria^4, 23, 24^. When localized at the inner mitochondrial membrane, its kinase domain likely faces the intermembrane space^4^. LETM1 is also a mitochondrial inner membrane protein with a single transmembrane domain. Its N-terminus is exposed to the intermembrane space, whereas its C-terminus is located in the matrix^18, 21^. Therefore, we hypothesized that PINK1 might phosphorylate the N-terminal region of LETM1 (residue 1–204). We searched for potential Thr phosphorylation sites of LETM1 in this region and identified two potential conserved motifs, Thr175 and Thr192, which were conserved in multiple species (Supplementary Fig. 2). Of these two sites, Thr192 was conserved in all seven species examined. We constructed and isolated bacterially expressed GST tagged N-terminus of LETM1-WT (residue 1–204, GST-N-LETM1) and mutations with the T175 or T192 site mutated singly to alanine. These constructs were analyzed by an in vitro kinase assay with His-tagged PINK1 with the N-terminus truncated to promote stability and kinase activity (deleted residues 1–111, His-∆N-PINK1). The results showed that PINK1 phosphorylated the WT and T175A but not T192A fragments, indicating that Thr192 of LETM1 is an appropriate candidate phosphorylation site for PINK1 (Fig. 1f). For further validation, we generated a custom phospho-antibody specific for phosphorylated LETM1 at Thr192 (pT192). We generated His-tagged LETM1 with the transmembrane portion deleted (His-∆TM-LETM1) to enhance recovery of bacterially generated LETM1. The pT192 antibody specifically recognized LETM1-WT phosphorylated by PINK1 but not unphosphorylated LETM1-WT or the T192A mutant incubated with PINK1 (Fig. 1g). Importantly, the pT192 phospho-antibody generated signal was blocked by pre-incubation with the corresponding phosphopeptide (p-peptide) antigen (CNGHTLpT^192^RRERR) (Fig. 1g, right) but not with the homologous non-p-peptide (CNGHTLTRRERR) (Fig. 1g, left). In addition, kinase-dead mutant K219M did not phosphorylate LETM1 (Supplementary Fig. 1d). Calf-intestinal alkaline phosphatase (CIP) treatment of phospho-LETM1 eliminated the phosphorylation signal (Fig. 1h). These data support the specificity of our generated pT192 antibody.

### Phospho-LETM1 is decreased in PINK1 KO and Q456X mutant

**Fig. 2:**
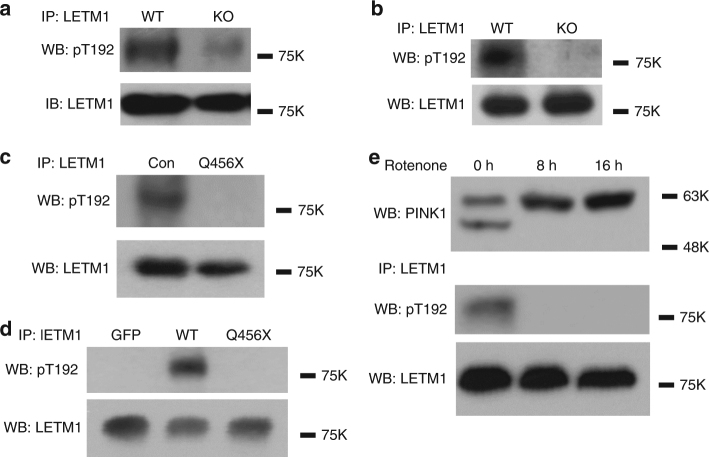
Deficiency or mutant PINK1 reduces phosphorylation of LETM1 at Thr192. **a**, **b** Proteins extracted from PINK1 WT or KO MEFs (**a**) or mouse brain (**b**) were subjected to IP with anti-LETM1, probed with pT192 and reprobed with anti-LETM1 by WB. **c** Proteins extracted from human fibroblast of control (Con) or a PINK1-Q456X patient (Q456X) were subjected to IP with anti-LETM1, probed with pT192 and reprobed with anti-LETM1 by WB. **d** HEK293 cells were transfected with Adtrack GFP control, AdPINK1-WT, and AdPINK1-Q456X mutant for 1 day. Endogenous LETM1 protein was isolated by IP with anti-LETM1, probed with pT192 and reprobed with anti-LETM1 by WB. **e** SH-SY5Y cells were treated with 25 µM rotenone for 8 or 16 h. Total cell lysates were either analyzed by WB with anti-PINK1, or subjected to IP with anti-LETM1, probed with pT192 and reprobed with anti-LETM1 antibodies by WB. All above experiments were replicated three times, respectively

To further confirm whether phosphorylation of LETM1 at Thr192 is dependent on endogenous PINK1, we utilized our pT192 phospho-antibody to detect phospho-LETM1 in PINK1 WT and knockout (KO) mouse embryo fibroblasts (Fig. 2a) and mouse brain tissues (Fig. 2b). Our results clearly showed that phosphorylation of LETM1 at Thr192 occurs in a PINK1-dependent manner. Similarly, we analyzed for pT192 signal in fibroblasts obtained from a PD patient expressing PINK1-Q456X, a nonsense mutant identified from PD patients with a partial deletion of the kinase domain resulting in a reduction in its kinase activity. LETM1 phosphorylation was dramatically reduced when compared to normal control individual (Fig. 2c). Conversely, expression of PINK1-Q456X in HEK293 cells also demonstrated reduced phosphorylation of LETM1 at Thr192 when compared to expression of PINK1 WT (Fig. 2d). Finally, PINK1 is stabilized at the outer mitochondrial membrane upon mitochondrial stress, topographically eliminating its ability to phosphorylate LETM1. We therefore tested whether mitochondrial damage induced by rotenone would reduce the pT192 LETM1 signal. Rotenone treatment stabilized full-length PINK1 as previously reported^25^. Importantly, this was associated with the absence of T192 phosphorylation (Fig. 2e). The absence of the T192 signal suggests that full-length PINK1 induced by mitochondrial stress previously reported to be stabilized at the outer membrane^5, 26^ does not phosphorylate LETM1. Taken together, this evidence strongly supports the PINK1 dependence of T192 LETM1 phosphorylation. Our data also support the notion that processed PINK1 at the inner mitochondrial membrane phosphorylates LETM1.

### Phospho-LETM1 increases the calcium transport in liposomes

**Fig. 3:**
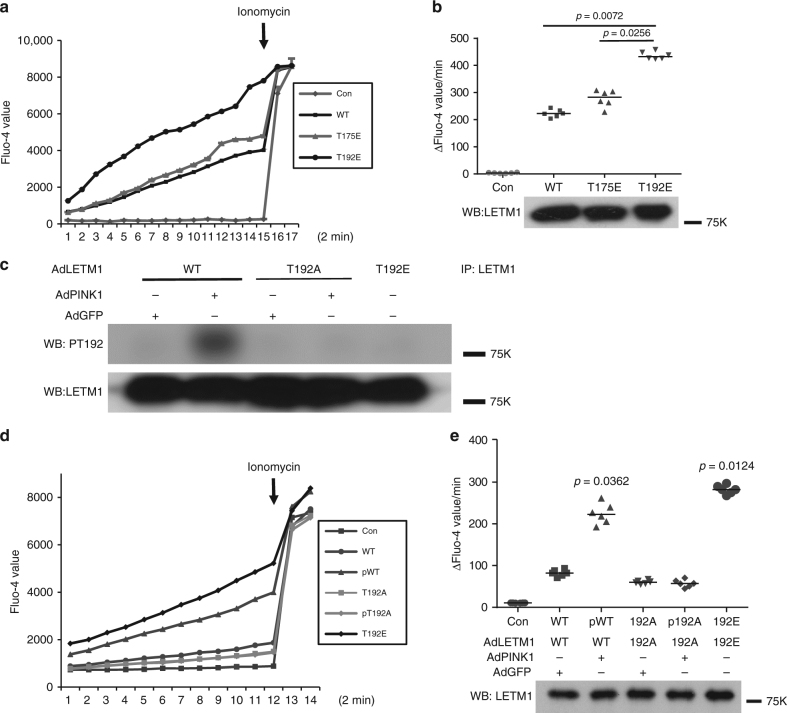
The effect of PINK1-mediated phosphorylation of LETM1 on liposomes calcium release activity in vitro. **a** About 1 µg purified bacterial full-length His-LETM1-WT, T175E, and T192E were incorporated into liposomes and subjected to calcium release assay. Control (Con) indicates no proteins incorporated. **b** (top panel) Quantification of calcium release activity measured by rate of calcium release and calculated as change of fluorescence unit (∆*F*). *n* = 6. (bottom panel) The liposome samples in **a** were analyzed by WB with anti-LETM1 antibody to show loading of proteins. **c** HEK293 cells were co-transfected with pAdtrack LETM1 (AdLETM1)-WT or T192A and Adtrack control GFP (AdGFP, WT, T192A) or PINK1 (AdPINK1, pWT, pT192A), and AdLETM1-T192E only for 1 day. The FLAG-LETM1 proteins were isolated by IP with anti-FLAG, probed with pT192 or anti-LETM1 by WB. The experiment was replicated three times. **d** HEK293 cells were co-transfected with AdLETM1-WT or T192A and AdGFP or AdPINK1, and AdLETM1-T192E only for 1 day. The FLAG-LETM1 proteins were isolated by IP with anti-FLAG and eluted by 3XFLAG peptide. The eluted proteins were subjected to calcium release assay using artificial liposomes. Control is without protein incorporated. **e** (top panel) Quantification of calcium release activity of **d** measured by rate of calcium release and calculated as change of fluorescence unit (∆*F*). *n* = 6. (bottom panel) The liposomes samples in **d** were subjected to WB with anti-LETM1 antibody to show similar loading of proteins

Previous evidence indicated that LETM1 regulates calcium transport in an artificial liposomal context^17, 27^. We therefore first assessed whether phosphorylation of LETM1 at Thr192 might affect its calcium transport activity in this defined setting. Initially, bacterially expressed full-length His-LETM1-WT, phospho-mimetic T175E, and T192E were subjected to a liposomal calcium release assay. As shown in Fig. 3a, b, the rate of calcium release regulated by LETM1-T192E was significantly higher than the rate of calcium release regulated by WT and T175E, suggesting the importance of T192 phosphorylation in calcium exchange activity. We also performed the converse with calcium uptake in liposomes. We showed similar results, i.e., that the calcium uptake activity of His-LETM1-T192E is higher than that of WT or T175E (Supplementary Fig. 3a, b). Next, we explored whether phosphorylation of LETM1 at Thr192 affects calcium transport in liposomes. We purified FLAG-LETM1 variants expressed in HEK293 cells coexpressed with PINK1 or GFP control by IP with anti-FLAG antibody. We confirmed that phosphorylation of LETM1-WT could be detected in cells contransfected with PINK1 but not with that of LETM1-T192A by our pT192 phospho-antibody (Fig. 3c). These eluted FLAG-tagged proteins were then incorporated into liposomes and assessed for calcium release assay. As shown in Fig. 3d, e, phospho-LETM1-WT(pWT) and LETM1-T192E showed an increased rate of calcium transport compared to other conditions (LETM1-WT, T192A without PINK1 (T192A), and T192A with PINK1(pT192A)). Similarly, these eluted FLAG proteins were also subjected to the liposomal calcium uptake assay. Similar to calcium release assay, the rate of calcium uptake by phospho-LETM1 and LETM1-T192E was significantly higher than the others (Supplementary Fig. 3c, d). Taken together, these data indicate that phosphorylation of LETM1 by PINK1 at Thr192 increases calcium handling in liposomes.

### LETM1-T192E rescues calcium transport in mitochondria

**Fig. 4:**
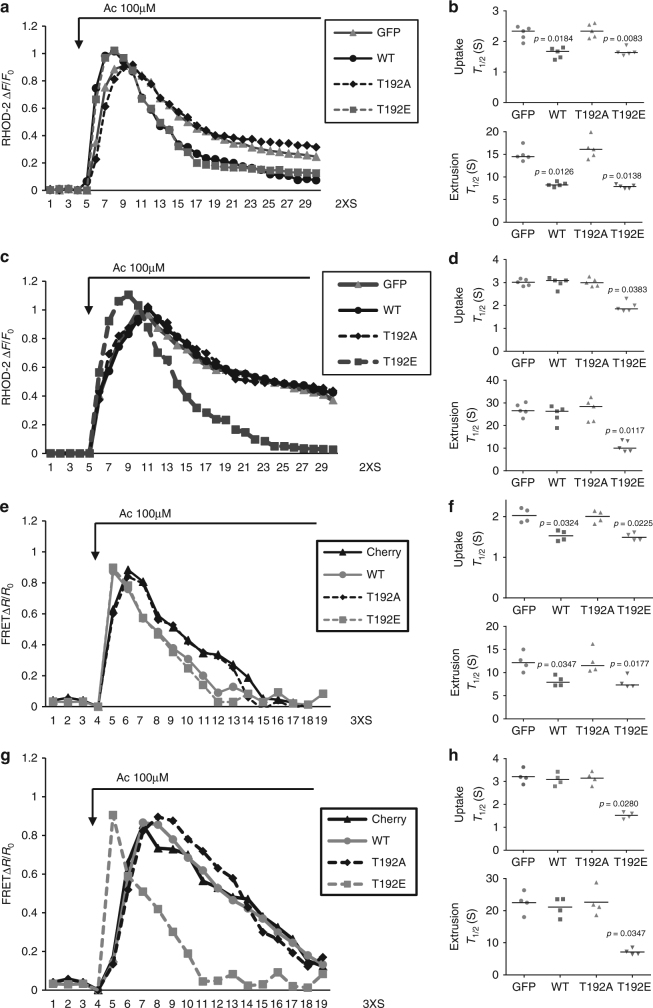
The effect of phospho-LETM1 at Thr192 on mitochondrial calcium transport in primary cortical neurons. **a** Representative fluorescence traces of mitochondrial Ca^2+^ imaging in neurons from PINK1 WT infected with adenovirus GFP, LETM1-WT, T192A, and T192E stimulated by Ac. **b** Quantification of the rate of calcium transport release activity in **a** measured by *T*_1/2_. *n* = 5. **c** Representative fluorescence traces of mitochondrial Ca^2+^ imaging in neurons from PINK1 KO infected with adenovirus GFP, LETM1-WT, T192A, and T192E stimulated by Ac. **d** Quantification of the rate of calcium transport release activity in **c** measured by *T*_1/2_. *n* = 5. **e** Representative traces of mitochondrial Ca^2+^ imaging in neurons infected with lenti-4mTG-TN-XXL from PINK1 WT co-infected with lenti-mitoCherry, LETM1-WT, T192A, and T192E stimulated by Ac. **f** Quantification of the rate of calcium transport in **e** measured by *T*_1/2_. *n* = 4. **g** Representative traces of mitochondrial Ca^2+^ imaging in neurons infected with lenti-4mTG-TN-XXL from PINK1 KO co-infected with lenti-mitoCherry, LETM1-3XFLAG-cherry-WT, T192A, and T192E stimulated by Ac. **h** Quantification of the rate of calcium transport in **g** measured by *T*_1/2_. *n* = 4

We next examined the role of LETM1 in mitochondrial calcium transport in a cellular environment. We initially utilized Rhod-2, which is cationic and preferentially accumulated into the mitochondria^28^, to observe the dynamic change of [Ca^2+^]_m_ in primary cortical neurons. First, we verified that Rhod-2 was co-localized with Mitotracker green (a mitochondrial marker) in both HEK293 cells and primary cortical neurons (Supplementary Fig. 4), indicating Rhod-2 mainly distributes in mitochondria under the conditions utilized. We next examined whether PINK1 affects [Ca^2+^]_m_ in neurons upon stimulation with neurotransmitter acetylcholine (Ac). The trace of [Ca^2+^]_m_ showed an earlier increase and later decrease with time indicative of calcium uptake and extrusion, respectively, in mitochondria. As shown in Supplementary Fig. 5a, b, both rates of [Ca^2+^]_m_ uptake and extrusion were compromised in PINK1 KO neurons. We next examined the effect of small interfering RNA (siRNA) to LETM1 (SiLETM1) in neurons and showed that two independent SiLETM1 constructs (S2 and S3) reduced the level of LETM1 (Supplementary Fig. 5c). Importantly, SiLETM1-S2 and S3 treatment both decreased [Ca^2+^]_m_ uptake and extrusion when compared to control siRNA (Supplementary Fig. 5d, e). To further examine the role of phosphorylation of LETM1, we virally expressed control GFP, LETM1-WT, T192A, or T192E mutants in PINK1 WT and KO neurons, respectively. First, we examined whether these viral vectors affect resting mitochondrial membrane potential (∆ψm) analyzed by TMRM in PINK1 WT or KO neurons. Our results show that although the ∆ψm was slightly lower in KO compared to WT neurons as previously reported^29^, the viral LETM1-WT, T192A, and T192E did not affect resting ∆ψm when compared to GFP (Supplementary Fig. 6a). For resting [Ca^2+^]_m_ analyzed by Rhod-2, there is no difference between PINK1 WT and KO neurons as previously reported^9^ and the viral LETM1-WT, T192A, and T192E did not affect the resting [Ca^2+^]_m_. (Supplementary Fig. 6b). However, the caveat to this observation is that differences in ∆ψm in PINK1 WT and KO neurons may affect Rhod-2 loading. As shown in Fig. 4a, b, LETM1-WT and T192E but not LETM1-T192A promoted [Ca^2+^]_m_ uptake and extrusion compared to GFP control in PINK1 WT neurons upon Ac stimulation. However, only LETM1-T192E but not WT and T192A rescued the compromised [Ca^2+^]_m_ uptake and extrusion in PINK1 KO neurons (Fig. 4c, d). These data indicated that PINK1-mediated phospho-LETM1 played a role in both mitochondrial calcium uptake and extrusion. Given that mitochondrial calcium uniporter (MCU) complex and mitochondrial Na^+^/Ca^2+^ exchanger (NCLX) also play a role in mitochondrial calcium uptake and release, respectively^30, 31^, we next examined whether there is potential cross-talk between LETM1 and MCU or NCLX. We performed Co-IP to check the interaction between LETM1 and MCU or NCLX in HEK293 cells. As shown in Supplementary Fig. 7, LETM1 did not interact with MCU (Supplementary Fig. 7a, b) or NCLX (Supplementary Fig. 7c, d).

Because of some potential issues with Rhod-2-based analyses as stated above, we further investigated the dynamic change of [Ca^2+^]_m_, employing the mitochondrial genetically encoded calcium indicator 4XmTG-TN-XXL^32^. We infected lentiviral 4XmTG-TN-XXL in neurons and showed that it distributed in mitochondria co-located with Mitored (Supplementary Fig. 8a). We also produced lenti-LETM1-3XFLAG-T2A-cherry variants co-infected with lenti-4XmTG-TN-XXL (Supplementary Fig. 8b). The expression of lenti-LETM1-3XFLAG-T2A-cherry variants were also validated by Western blotting (Supplementary Fig. 8c). As shown in Fig. 4e, f and Supplementary Fig. 9, LETM1-WT and T192E but not LETM1-T192A promoted [Ca^2+^]_m_ uptake and extrusion compared to GFP control in PINK1 WT neurons infected with lenti-4XmTG-TN-XXL upon Ac stimulation. However, only LETM1-T192E but not WT and T192A rescued the compromised [Ca^2+^]_m_ uptake and extrusion in PINK1 KO neurons (Fig. 4g, h). Taken together, these data support a model in which phosphorylation of LETM1 at T192 regulates [Ca^2+^]_m_ and defects in calcium management observed with PINK1 loss can only be rescued by the LETM1-T192E phosphomimic.

Finally, it has been reported that loss of PINK1 compromises NCLX and lead to impaired mitochondrial Ca^2+^ extrusion^33^. To investigate whether NCLX and LETM1 function in the same pathway to regulate Ca^2+^ extrusion in cortical neurons, an NCLX inhibitor CGP 37157 was employed. This inhibitor delayed [Ca^2+^]_m_ extrusion. In the presence of this NCLX inhibition, expression of LETM1 still increased [Ca^2+^]_m_ extrusion (Supplementary Fig. 10a, b). Conversely, KD of LETM1 by siRNA S2 or S3 additionally delayed [Ca^2+^]_m_ extrusion in Ac-stimulated neurons in the presence of the NCLX inhibitor CGP 37157 (Supplementary Fig. 10c, d). Taken together, these data indicated that LETM1 and NCLX are involved in [Ca^2+^]_m_ extrusion but likely in an independent manner.

### Phospho-LETM1 protects against neuronal death

**Fig. 5:**
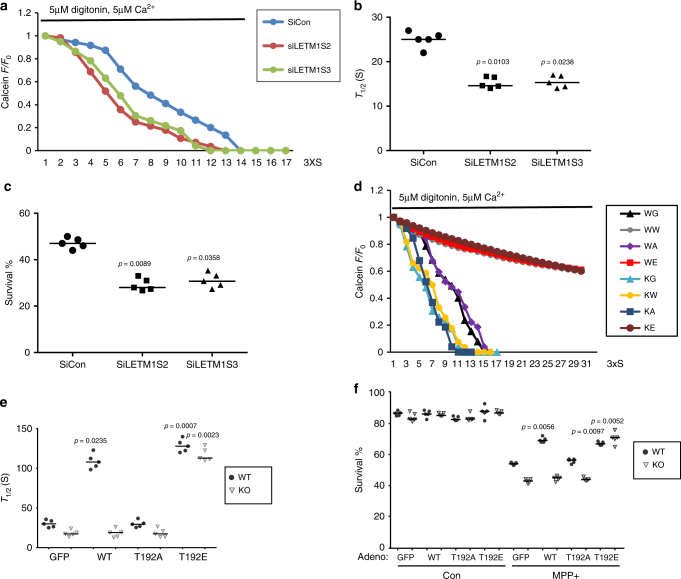
The protective role of phospho-LETM1 at Thr192 in mPTP opening and neuronal death. **a**, **b** Primary cortical neurons were transfected with control SiCon, SiLETM1-S2, and -S3 and then were processed to calcium-induced mPTP opening assay. **a** Representative average calcein fluorescence traces of neurons are presented. **b** Half-life time was calculated for groups in **a**. *n* = 5. **c** Survival assay was performed in siRNA-treated neurons. *n* = 5. **d** Cortical neurons from PINK1 WT were infected with lenti-mitoCherry (WC), LETM1-Cherry WT (WW), T192A (WA), or T192E (WE) or from PINK1 KO were also infected with lenti-mitoCherry (KC), LETM1-Cherry WT (KW), T192A (KA), or T192E (KE) for 5 days and then were processed to calcium-induced mPTP opening assay. Representative average calcein fluorescence traces of neurons are presented. *n* = 5. **e** Half-life time was calculated for groups in **d**. *n* = 5. **f** Primary cortical neurons from PINK1 WT and KO were infected with adenovirus expression GFP, LETM1-WT, T192A, or T192E and then treated with 75 µM MPP+ or saline for 24 h at DIV3. Neuronal survival was evaluated after MPP+ or saline treatment. *n* = 5

**Fig. 6:**
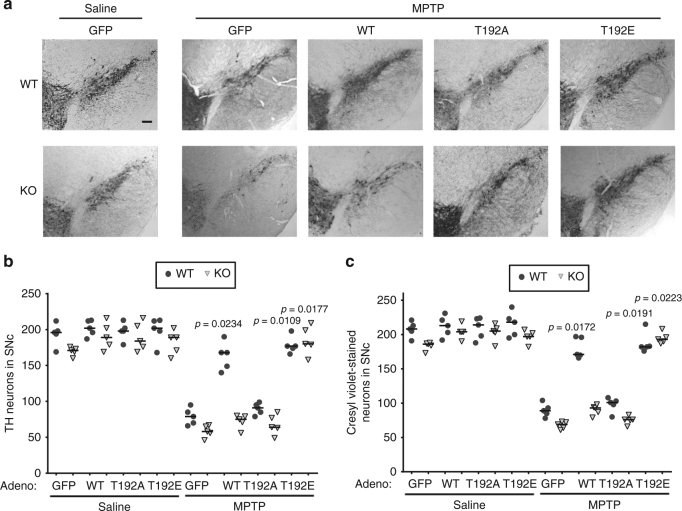
The protective role of phospho-LETM1 at Thr192 in MPTP-injected mice. **a** Representative images of the ipsilateral side in PINK1 WT and KO mice injected with corresponding AdGFP, LETM1-WT, T192A, and T192E viruses as indicated with saline or MPTP injected 1 week later. Scale bar: 100 µm. **b** Quantification of TH-positive dopaminergic neurons from ipsilateral sides in the indicated groups. *n* = 5. **c** Quantification of cresyl violet-stained neurons at the ipsilateral sides from the indicated treatment groups. *n* = 5

Our data support the notion that PINK1-mediated phosphorylation of LETM1 plays an important role in [Ca^2+^]_m_ transport. Deficient PINK1 would lead to [Ca^2+^]_m_ mishandling and overload, which can induce mitochondrial permeability transition pore (mPTP) opening and contribute to neuronal death^11, 12^. We therefore investigated the role of PINK1-mediated LETM1 phosphorylation in mPTP opening and neuronal death/degeneration. First, we showed that the level of LETM1 protein was not significantly changed in neurons with MPP^+^ treatment (Supplementary Fig. 11a, b) and knockdown of LEMT1 by SiLETM1-S2 and -S3 sensitized cultured neurons to calcium-induced mPTP opening (Fig. 5a, b) and MPP^+^ treatment (Fig. 5c). Second, expression of LETM1-WT and LETM1-T192E but not LETM1-T192A or GFP control in PINK1 WT neurons protected against calcium-induced mPTP opening (Fig. 5d, e) and MPP^+^ challenge (Fig. 5f). However, only expression of LETM1-T192E in PINK1 KO neurons resulted in protection against calcium-induced mPTP opening (Fig. 5d, e) and MPP^+^ challenge (Fig. 5f). Similar findings were observed in an in vivo MPTP model. We examined the effects of expression of adenoviral GFP, LETM1-WT, T192A, and T192E in DA neuronal loss in MPTP injected mice. We verified the expression of LETM1 and its mutants in the substantia nigra pars compacts (SNc) of mice by WB (Supplementary Fig. 12a) and immunofluorescence (Supplementary Fig. 11b) after adenoviral injection. In the saline-injected control group, expression of adeno-LETM1-WT and T192A and T192E did not lead to significant neuronal death in the SNc of WT and KO mice (Fig. 6a, b). Expression of LETM1-WT and T192E but not T192A significantly protected DA neurons from loss compared to the GFP control in PINK1 WT mice, whereas only expression of LETM1-T192E but not WT and T192A protected DA neurons in PINK1 KO mice (Fig. 6a, b). Consistent with this assessment of survival by TH immunostaining, cresyl violet staining also showed similar results (Fig. 6c). Taken together, our in vitro and in vivo data suggests that phosphorylation of LETM1 by PINK1 plays an important role in calcium homeostasis, which is associated with protection of neurons from exogenous stress.

## Discussion

In this study, we demonstrate that PINK1 directly interacts with LETM1 and phosphorylates LETM1 at Thr192. Phospho-LETM1 at Thr192 not only increases the rate of calcium release/uptake in liposomes but also facilitates calcium transport in intact mitochondria of neurons. PINK1 KO and PD-associated mutant PINK1-Q456X dramatically decreases phospho-LETM1, resulting in mitochondrial calcium mishandling and neuronal vulnerability. Expression of LETM1-T192E, a phospho-mimetic mutant, but not LETM1-WT rescues the mitochondrial calcium mishandling in PINK1 KO neurons. Expression of LETM1-WT and T192E protected MPP^+^/MPTP-induced neuronal death in PINK1 WT mice, while only LETM1-T192E protects neurons in PINK1 KO mice. Interestingly, LETM1-WT could increase calcium transport in artificial liposome but expression of LETM1-WT (or 192A) did not rescue calcium transport in mitochondria and did not prevent neuronal death in PINK1 KO mice. The reason for this is unclear but may reflect a differential response in artificial liposome vs. mitochondrial context. For example, LETM1-WT is present endogenously in neurons, which may impact the response of exogenously expressed LETM1 constructs. Nonetheless, our data shed light on an important mechanism by which PINK1-mediated LETM1 phosphorylation plays a critical role in mediating mitochondrial calcium transport associated with neuronal survival. Deficient or mutant PINK1 reduces LETM1 phosphorylation and consequently results in mitochondrial calcium mishandling.

Mitochondrial Ca^2+^ transport is complex and cell type dependent^34^. In this regard, the MCU complex plays an important role in mitochondria calcium uptake. However, the MCU complex, due to its low Ca^2+^ affinity, demands high local Ca^2+^ concentrations above the micromolar level, which are achieved by a close proximity to ER–mitochondria contact sites and regulates ER/mitochondria Ca^2+^ transfer. Other than the MCU complex, mitochondrial Ca^2+^ uptake via rapid mode (RaM) is an alternative/additional pathway suggested to work when [Ca^2+^]_c_ increased only above 200 nM^26, 31, 35^. Calcium signaling in neurons not only involves ER but is also dominated by Ca^2+^ influx through voltage- and ligand-gated ion channels^36^. Ca^2+^ could be directly transported into mitochondria by non-MCU mechanisms and LETM1 may play a critical role in this mitochondrial calcium uptake pathway. In support of this, our data show that Ac stimulation can activate Ca^2+^ influx from extracellular calcium sources and results in PINK1- and LETM1-dependent mitochondrial Ca^2+^ management. It is important to note that there is some controversy on whether LETM1 acts directly as a Ca^2+^/H^+^ exchanger. Accordingly, we cannot rule out that the observed calcium regulatory effects by PINK1 may be indirectly reliant on LETM1, at least in a cellular context. In this regard, LETM1 has also been suggested to regulate K^+^^37^. In addition, mitochondrial Na^+^/Ca^2+^ exchanger (NCX) also mediates [Ca^2+^]_m_ extrusion and it has been recently reported that the activity of a mitochondrial Na^+^/Ca^2+^ exchanger NCLX is impaired in PINK1 deficiency, which can be rescued by PKA^33^. However, our data support that LETM1 and NCLX are involved in [Ca^2+^]_m_ extrusion in a manner that is likely independent. Nonetheless, our data strongly support a model by which PINK1 regulation of LETM1 is critical for neuronal death induced by mitochondrial stress both in vitro and in vivo.

Neurodegenerative disorders including PD exhibit dysregulation of calcium homeostasis due to excitotoxicity, perturbed energy metabolism and oxidative stress, and compromised cellular calcium-regulating systems^38^. It has been suggested that adult DA neurons engage L-type channels permeable to calcium to drive pacemaking, leading to elevated intracellular calcium and neuronal vulnerability^39^. In stressed neurons, extracellular calcium enters the cell via calcium permeable channels (i.e., glutamate receptors) elevating [Ca^2+^]_C_ and [Ca^2+^]_m_^38^. Accordingly, one interesting model is that PINK1-mediated phosphorylation of LETM1 at Thr192 could facilitate [Ca^2+^]_m_ extrusion to prevent sustained [Ca^2+^]_m_ accumulation and protect neurons from stress and death. At least in models of mitochondrial stress, this appears to hold true. However, an important caveat is that MPP^+^/MPTP models of degeneration may not accurately reflect the true age-dependent processes in human PD. In this regard, the study of degenerative processes in model rodent systems is hampered by the lack of robust DA degeneration with respect to genetic forms of PD like with PINK1 loss^40^. However, it is intriguing to speculate that because of the proposed pacemaking capacity of DA neurons, a PINK1-LETM1 regulatory mechanism may be particularly critical in PD. In this model (Supplementary Fig. 13), we hypothesize that mutant or deficient PINK1 results in decreased phosphorylation of LETM1, consequently accelerating [Ca^2+^]_m_ mishandling and overload, rendering neurons vulnerable to stress.

## Methods

### Antibodies

The following antibodies were used: anti-FLAG (Sigma, Cat: F3165, dilution 1:10,000), anti-Myc (Abcam, Ab9106, 1:2500), anti-Phosphothreonine (pThr, Thermo Fisher, 71-8200, 1:2500); anti-Phosphoserine (pSer, Thermo Fisher, 61–8100, 1:2500); anti-LETM1 (Santa Cruz, mouse sc-514136, 1:2500 or Novus Biologicals, rabbit NBP1-33433, 1:10,000). Anti-PINK1 (Abgent, mouse AM6406a, 1:5000 and Novus Biologicals, rabbit BC100–494, 1:5000), anti-Tyrosine hydroxylase (TH, EMD Millipore, AB152, 1:1000), anti-MCU (Cell Signaling, 14997, 1:10,000), anti-NCLX (Abcam, ab136975, 1:5000). Special custom phospho-LETM1 at Thr192 (pT192) polyclonal antibody was generated and purified from a rabbit immunized with carrier protein-conjugated phosphopeptide, CNGHTLpT^192^RRERR (residues 187–197 of human LETM1, NP_036450.1), using standard protocols by Biogenes (Berlin, Germany, dilution 1:2500).

### Plasmid construction and virus production

The full open reading frame complimentary DNA clone of human LETM1 was purchased from Origene and was cloned into pCMV-3Tag-2a (Myc-Tag) and sequence was verified by DNA sequencing. LETM1 mutants were generated using QuickChange Site-Directed Mutagensis Kit (Stratagene, USA). LETM1 variants were engineered into the pGEX4T-1 or pET28a vector for preparation of GST- or His-tag fusion proteins^41^. LETM1 variants and PINK1 were cloned to pAdtrack-CMV with 3XFLAG at C-terminus for preparation of recombinant adenovirus as previously described^42^. The viral constructs expressing 3XFLAG-LETM1 variants and GFP are controlled by separate CMV promoters. The adenoviruses were produced using pAdEasy system as previously described^43^.

### Bacterial protein purification and in vitro kinase assay

For production of bacterial recombinant protein, transmembrane domains in amino-acid residues 1–111 of PINK1 were deleted by plasmid cloning and designated ΔN-PINK1. Only N-terminus (amino-acid residues 1–204) of LETM1 without transmembrane domains and C-terminus were produced by cloning and designated N-LETM1. The transmembrane domain (TM; amino-acid residue 205–227) of human LETM1 were deleted and designated ∆TM-LETM1. All GST- or His-tagged fusion proteins were expressed in *Escherichia coli* and purified using Sepharose 4B agarose (GE Healthcare, USA) or Ni-NTA Agarose (Qiagen Inc, Canada) respectively, as per manufacturer’s instruction. The PINK1 kinase assay with LETM1 variants was performed as previously described^44, 45^. Briefly, 10 µg GST-N-LETM1 or His-∆TM-LETM1 variants with 2 µg His-ΔN-PINK1 was incubated at 30 °C for 6 h in 40 µl of reaction buffer containing 50 mM Tris-HCL (pH 7.4), 10 mM MgCl_2_, 2 mM MnCl_2_, 0.5 mM Calcium, 2 mM DTT and 0.1 mM ATP. The reactions were subjected to WB with pSer, pThr, or pT192 antibodies.

### Immunoprecipitation

Cells or samples were harvested in lysis buffer (50 mM Tris-HCl (pH 7.4), 100 mM NaCl, 2 mM EDTA, 1 mM DTT, 1% Triton X-100, and 0.1% SDS) supplemented with protease inhibitors. IPs were performed through incubation of 2 µg antibodies with lysates (2 mg protein) at 4 °C for 2 h followed by incubation with protein A beads (Sigma, USA) at 4 °C for 2 h. The washed samples were analyzed by Western blotting using TrueBlot secondary antibodies (Rockland Immunochemicals Inc). Some IPed FLAG-tagged proteins were eluted with 3XFLAG peptide (Sigma) according to the manufacturer’s instructions.

### Artificial liposomes

The calcium transport activity of LETM1 was measured as calcium released from artificial liposomes as described previously with minor modification^17, 27, 46^. Liposomes were reconstituted by mixing 100 mg/ml asolectin, 10 mg/ml cardiolipin in solubilization solution (Tris 50 mM, pH 7.4, 2% Triton-100, 2 mM calcium, EDTA 0.5 mM) and sonicated until the solution was clear. Combined with varied purified LETM1 proteins, the liposomes were incubated at room temperature for 1 h. Detergent was then removed by passing Amberlite XAD-2 (Sigma) columns three times. Extraliposomal solutions were changed by passing eight times through Sephadex G-25 (GE Healthcare) columns swelled with TE buffer (Tris 50 mM/EDTA 0.5 M). The free [Ca^2+^] was measured (excitation at 488 nm and emission at 530 nm) in a plate reader by loading 10 μM Fluo-4 pentapotassium salt (Invitrogen). The calcium uptake assay in liposome was also performed. Liposomes were reconstituted by mixing 20 mg/ml 3:1 POPE (850757P; Avanti Lipids Polar)/POPG (840457P, Avanti Lipids Polar) in solubilization solution (Tris 50 mM, pH 7.4, 2% Triton-100, EDTA 1 mM). The rest of the procedure is similar to the calcium release assay. Extraliposomal solutions contained Tris 50 mM/EDTA 0.5 mM. Ca^2+^ was added to extraliposomal solutions to create the initial 750 nM free [Ca^2+^] (CaCl_2_ 0.47 mM/EDTA 0.5 mM) and then the free [Ca^2+^] was measured (excitation at 488 nm and emission at 530 nm) in a plate reader by loading 10 μM Fluo-4 pentapotassium salt (Invitrogen). [Ca^2+^] was calculated using WEBMAXC (http://www.stanford.edu/~cpatton/webmaxcS.htm).

### Neuronal cultures with survival assay and RNA interference

Primary culture of mouse cortical neurons was carried out as described previously^47, 48^. For infection, 24 h after initial plating the cortical neurons were infected with adenovirus (MOI 100). Two days after infection, cortical neurons were subjected to survival determination following exposure to 75 μM MPP^+^ for 24 h. For survival of infected neurons, GFP-positive neurons were assessed for nuclear integrity as determined by Hoechst 33258 (0.5 ng/ml) staining. Neurons with punctate or condensed nuclei were assessed as dead. Survival was expressed as the percentage of live cells to total cells. SiRNA to mouse LETM1 was produced utilizing the Silencer siRNA Construction Kit (AM1620, Thermo Fisher Scientific) as per manufacturer’s instruction. The sequences of sense strand siRNA to LETM1 and scramble control are: S1 (5′-UGAGGAAAUCAUGC-GUUUU-3′); S2 (5′-GGAGGAGAUUGACAUCCUC-3′); S3 (5′-GCAAAUCAAGCACA-UUCCA-3′); scramble control siRNA (5′-GUAGCACGCGUA ACUGUCU-3′). Neurons were transfected with 30 pmol/well (24 well plate) siRNA to mouse LETM1 (SiLETM1) using Lipofectamine 2000 (Invitrogen, USA) for 2 days and re-transfected once at 3 days.

### TMRE staining for mitochondrial membrane potential

Cortical neurons were loaded with 200 nM tetramethylrodamine methylester (TMRM; T669; Thermo Fisher) in imaging buffer (20 mM HEPES, 135 mM NaCl, 5 mM KCl, 1 mM CaCl_2_, 30 mM d-glucose, 10 mM succinate, pH 7.4) for 30 min at room temperature, and the dye was present during the imaging experiment analyzed by a Zeiss 510 meta confocal microscope.

### Mitochondrial Ca^2+^ imaging in individual neurons

Mitochondrial Ca^2+^ imaging in individual primary cortical neurons employed Rhod-2 dye, which is cationic and preferentially accumulated in the mitochondria^28^. Cortical neurons at DIV3–4 on coverslip infected with adenovirus were loaded with Rhod-2-AM (Invitrogen) 1 µM and 0.01% (w/v) pluronic acid in imaging buffer (20 mM HEPES, 135 mM NaCl, 5 mM KCl, 1 mM CaCl_2_, 30 mM d-glucose, 10 mM succinate, pH 7.4) at room temperature for 30 min. Subsequently, cells were washed with imaging buffer and further incubated for 60 min at 37 °C for de-esterification of dye. Mitochondrial Ca^2+^ imaging experiments were performed in the imaging chamber (Warner Instruments) at 37 °C with Zeiss AxioObserver. D1 Microscope with ×40 objective. Rhod-2 was excited at 543 nm, and emission measured at 580 nm. The increased mitochondrial calcium was stimulated by 100 µM Ac. The images were captured in a time lapse model at 2s intervals for 2 min and neurons were stimulated at the 10th frame of recording. Relative increase in fluorescence intensity was normalized by fluorescence at the resting condition (Δ*F*/*F*_0_). Data were generated and analyzed with Zeiss microscope Axiovision rel 4.8 software. The rate of uptake or extrusion of mitochondrial calcium was expressed as *T*_1/2_ calculated by Logarithmic (LOG) function formatted as *T*_1/2_ = Exp[(*Y*_1/2_–*b*)/*a*] in Microsoft Excel 2010. Data were presented as the traces of average values for all data points generated from a minimum of 20 neurons per experiment and at least five independent experiments.

### FRET-based mitochondrial calcium imaging

TN-XXL is a genetically encoded FRET calcium sensor and based on the calcium binding protein troponin C linking cyan fluorescent protein (CFP) and Citrine Cp174 fluorescent protein (one YFP). We produced a mitochondrial targeted 4XmTG-TN-XXL construct, where four mitochondrial targeting sequences (mTGs) derived from the subunit VIII of human cytochrome *c* oxidase were fused with the N-terminal of TN-XXL. 4XmTG-TN-XXL (kindly provided by Dr. Oliver Griesbeck, Max Planck Institute of Neurobiology, Martinsried, Germany) was subcloned into lentiviral pLVX-AcGFP1-N1 vector (Clotech, Cat No: 632154) with deletion of GFP. We also generated the mito-targetted PLVX-4XmTG-Cherry as control and PLVX-LETM1-3XFLAG-T2A-Cherry WT, T192A, and T192E constructs with deletion of GFP. T2A is a self-cleaving 2A peptide^37^. Lentiviruses were generated by PLVX, pPAX2, and pMD2.G system. Lenti-4XmTG-TN-XXL were co-infected with lenti-4XmTG-Cherry, LETM1-3XFLAG-T2A-Cherry WT, T192A, and T192E in neurons (MOI 10) at DIV 1 for 5–7 days before calcium imaging.

For FRET-based mitochondrial calcium imaging, all FRET microscopic observations were performed on a Zeiss 510 meta confocal microscope at 37 °C. Donor (CFP) was excited at 458 nm and detected in a bandwidth of 470–500 nm (CFP channel), whereas the excitation at 514 nm and emission at 505–550 nm were used for detecting acceptor (YFP) (YFP channel). For FRET, the excitation was at 458 nm and detection at 505–550 nm (FRET channel). CFP and FRET emission channels were recorded simultaneously by passing through a long-pass dichroic mirror (515 nm). The images were captured in a time-lapse model at 3s intervals for 2 min and neurons were stimulated at the 5th frame of recording by 100 µM Ac. Images were analyzed using Image J software. The Intensity of FRET/CFP was calculated as FRET ratio. Relative increase in FRET ratio was normalized by the resting condition (Δ*R*/*R*_0_). The rate of uptake or extrusion of mitochondrial calcium was expressed as *T*_1/2_ calculated by LOG function formatted as *T*_1/2_ = Exp[(*Y*_1/2_–*b*)/*a*] in Microsoft Excel 2010. Data were presented as the traces of average values for all data points generated from a minimum of 10 neurons per experiment and at least four independent experiments.

### Measurement of the mPTP opening

mPTP opening was assessed by the quenching of calcein fluorescence with cobalt^30^. PINK1 WT or KO neurons were loaded with Calcein-AM (1 μM, Molecular Probes) at 37 °C for 15 min and CoCl_2_ (2 mM, Sigma) was added for further 30 min. Neurons were washed and then permeabilized with 5 μM digitonin in 5 μM Ca^2+^ in imaging buffer (20 mM HEPES, 135 mM NaCl, 5 mM KCl, 30 mM d-glucose, 10 mM succinate, pH 7.4) to induce mPTP opening. Images were captured with the Zeiss AxioObserver. D1 Microscope in a time-lapse model at 3s intervals for 2 min. Relative calcein fluorescence intensity was normalized to the resting condition (Δ*F*/*F*_0_) and *T*_1/2_ was calculated. Data were generated and analyzed with the Zeiss microscope Axiovision rel 4.8 software.

### Animals with viral delivery and MPTP administration

PINK1-deficient mice (maintained on a C57BL/6 background) and wild-type littermates were generated by breeding their heterozygous counterparts. Genotyping information was reported previously^49^. Mice were housed two to five animals per cage with a 12-h light/dark cycle (lights on from 0700 to 1900 h) at constant temperature (23 °C) with ad libitum access to food and water. All animal experiments conformed to the guidelines set forth by the Canadian Council for the Use and Care of Animals in Research (CCAC) and the Canadian Institutes for Health Research (CIHR) and with approval from the University of Ottawa Animal Care Committee.

We have previously shown that adenoviruses were targeted unilaterally to DA neurons of SNc from the striatum by retrograde transport using a well-established adenoviral-mediated gene delivery approach^14^. About 52 male PINK1 WT and 52 male KO mice of 8–12 weeks were randomly allocated to four groups, respectively. A single unilateral injection of each virus expressing GFP, LETM1-WT, T192A, or T192E (2 µl, 1 × 10^7^ particles per µl) was delivered to the right striatum (0.5 mm rostral, 2.2 mm right of bregma, and 3.4 mm below the skull surface) of 8-week-old male mice using a syringe pump system 7 days before MPTP administration. To examine the viral expression in TH neurons, the samples extracted from SNc of post 7 days virus-injected mice were subjected to Western blotting. Coronal sections (14 µm thickness) of the ventral midbrain were double labeled using specific primary antibody to GFP (Abcam) or TH (Millipore).

Mice received one intraperitoneal injection of MPTP·HCl per day (30 mg/kg, Sigma, USA) for 5 consecutive days^50–53^. Control mice received an equivalent volume of 0.9% saline. Assessment of dopamine neuron survival was performed blindly 2 weeks after the start of the MPTP injection by immunohistochemical analyses.

### Immunohistochemistry and immunofluorescence

Mice were perfused transcardially and brains were fixed in 4% paraformaldehyde and cryoprotected as previously described^54^. Free floating serial coronal sections (14 µm thickness) of the ventral midbrain were collected. Sections were then incubated with TH antibody (1:1000) for 24 h at 4 °C. Immunoreactivity was visualized by using an avidin–biotin complex peroxidase/3,3′-diaminobenzidine (DAB) reaction. For double-labeling immunofluorescence, samples were double labeled with primary antibody to GFP (Abcam) or TH (Millipore) and visualized using either Alex-488-conjugated anti-rabbit IgG (1:200) or Alex-594-conjugated anti-mouse IgG (1:200).

### Quantification of dopaminergic neuronal loss

The number of DA (TH positive) neurons was only counted from the sections in the region containing the medial terminal nucleus (MTN) because this region has been previously shown to express the highest level of virus-mediated gene expression after intrastriatal infection^50^. We also used the MTN as a landmark to evaluate consistent levels of SNc. Neurons in the ipsilateral and contralateral side to the viral injection were assessed as previously described^50^. At least three sections per animal were analyzed. In parallel, cresyl violet staining was performed to validate determination of nigral counts as previously described^50^. The anatomical localization of SNc was referenced by TH staining.

### Statistical analysis

Statistical differences between multiple groups of data were all analyzed with nonparametric Kruskal–Wallis one-way ANOVA test using GraphPad Prism v7.03 program. The data are presented as median. Exact *p*-value is indicated in the graph if *p* < 0.05.

### Data availability

All data generated or analyzed during this study are either included in this published article (and its Supplementary Information files) or available from the authors.

## Electronic supplementary material

Supplementary Information
