# Supplementary material for: PINK1-mediated phosphorylation of LETM1 regulates mitochondrial calcium transport and protects neurons against mitochondrial stress
Source: Nat Commun. 2017 Nov 9;8:1399. doi: 10.1038/s41467-017-01435-1 (PMC5680261; doi:10.1038/s41467-017-01435-1)

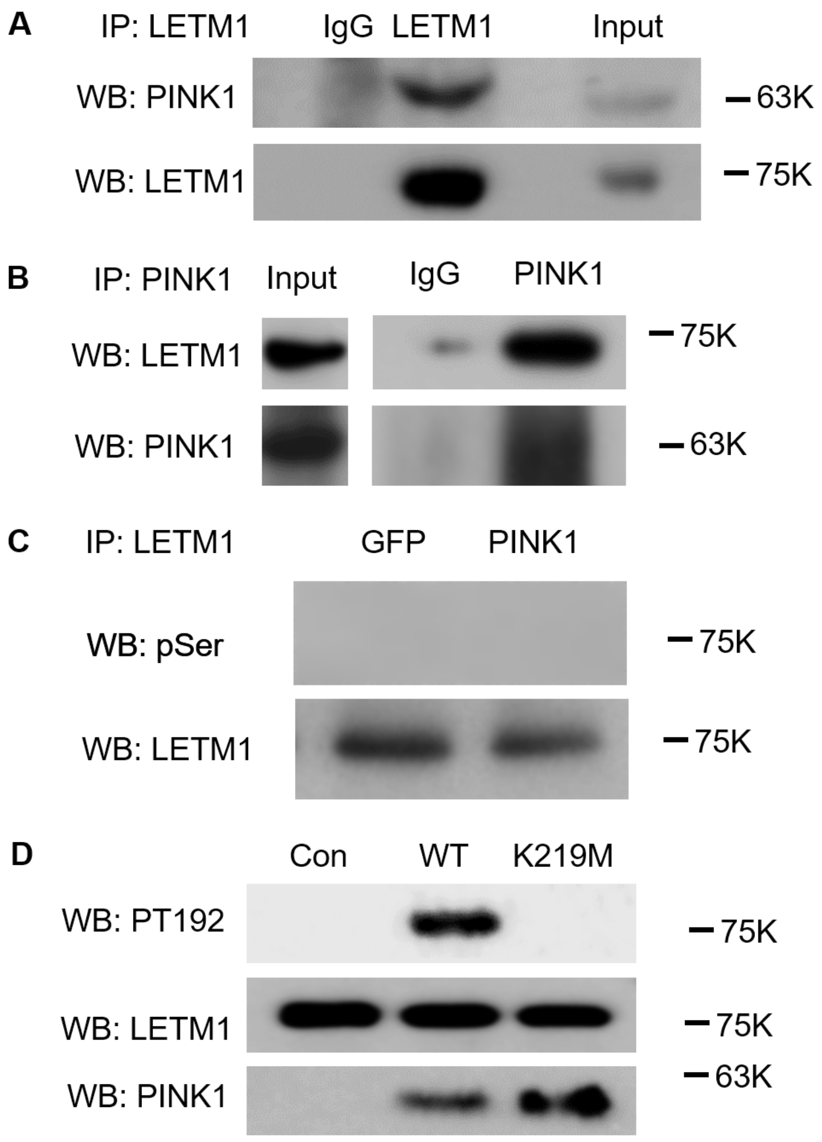


**Figure. S1.** (A) Endogenous PINK1 interacts with LETM1 in human SH-SY5Y neuroblastoma cells. SH-SY5Y cell lysate was subjected to IP with control IgG or anti-LETM1. The isolated proteins were probed with anti-PINK1 and reprobed with anti-LETM1. (B) Conversely to (A), the isolated proteins by IP with control IgG or anti-PINK1 were probed with anti-LETM1 and reprobed with anti-PINK1. (C) PINK1 does not phosphorylate LETM1 at Serine *in vivo*. HEK293 cells were transfected with GFP or pAdtrack-PINK1 for one day. Pan-LETM1 was pulled down by IP with anti-LETM1 and probed with phospho-Serine antibody (pSer) and reprobed with anti-PINK1. (D) Bacterial His-ΔN-LETM1 WT were subjected to *in vitro* kinase assay with His-ΔN-PINK1 WT and kinase dead mutant K219M and probed with pT192 phospho-antibody. Samples were reprobed with anti-LETM1 for loading. All above experiments were replicated 3 times.

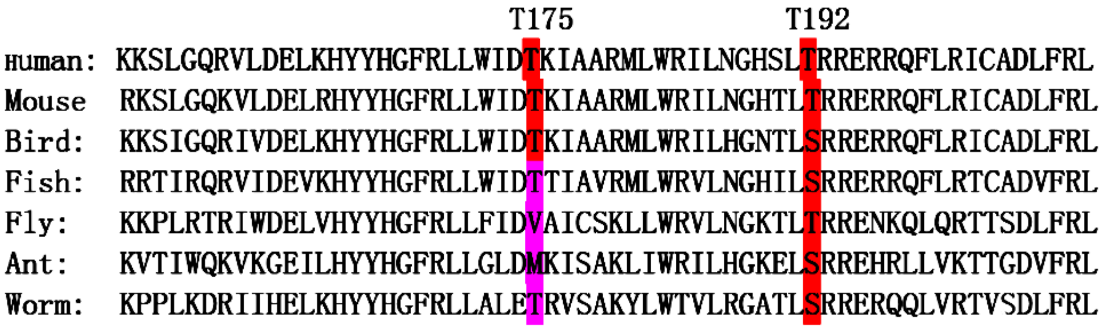


**Figure. S2.** The sequence alignment of N-terminal LETM1 (residue 150-204) in different species.

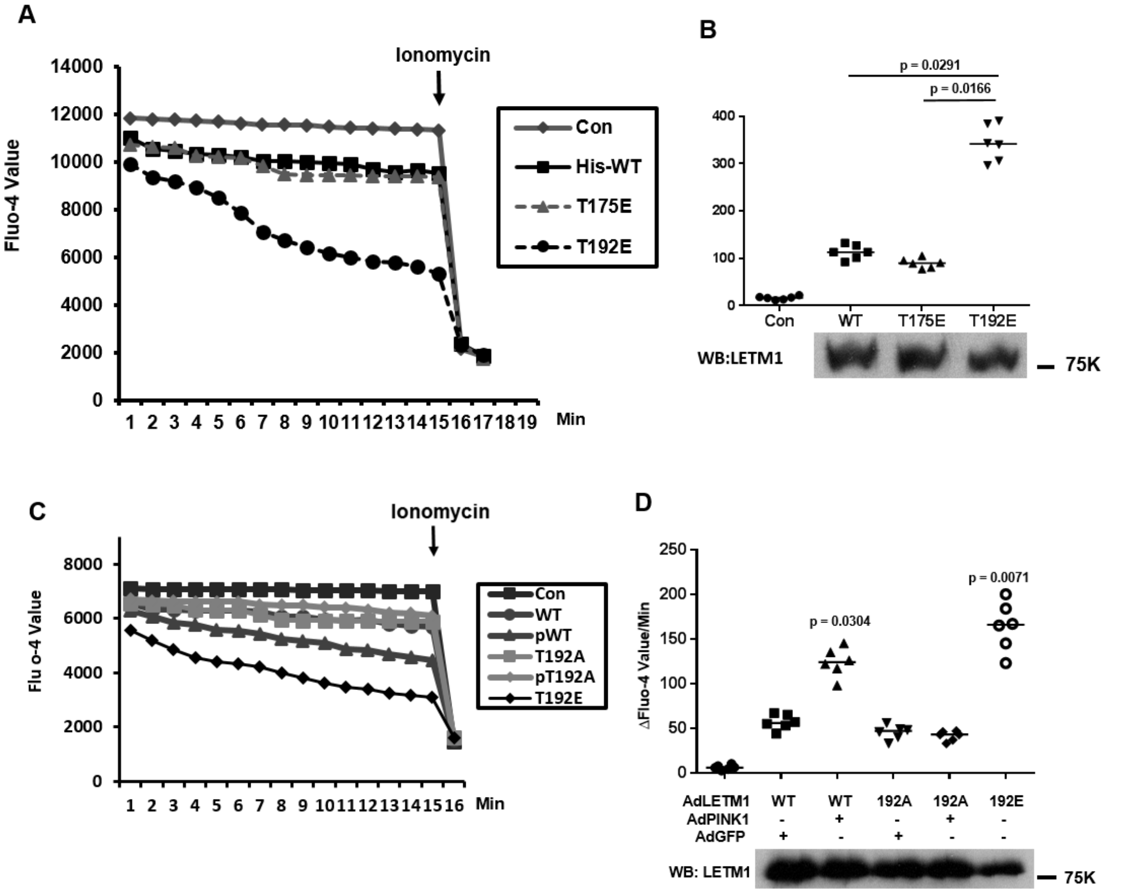


**Figure. S3**. The effect of PINK1-mediated phosphorylation of LETM1 on liposomes calcium uptake activity *in vitro*. (A) 1 ug purified bacterial full length His-LETM1-WT, T175E and T192E were incorporated into liposomes and subjected to calcium uptake assay. Control (Con) indicates no proteins incorporated. (B) (Top panel) Quantification of calcium release activity measured by rate of calcium release and calculated as change of fluorescence unit ($\Delta$F). n = 6. (Bottom panel) The liposome samples in (A) were analyzed by WB with anti-LETM1 antibody to show loading of proteins. (C) HEK293 cells were co-transfected with AdLETM1-WT or T192A and AdGFP or AdPINK1, and AdLETM1-T192E only for one day. The FLAG-LETM1 proteins were isolated by IP with anti-FLAG and eluted by 3XFLAG peptide. The eluted proteins were subjected to calcium uptake assay using artificial liposomes. Control is without protein incorporated. (D) (Top panel) Quantification of calcium release activity of (D) measured by rate of calcium uptake and calculated as change of fluorescence unit ($\Delta$F). n = 6. (Bottom panel) The liposomes samples in (D) were subjected to WB with anti-LETM1 antibody to show similar loading of proteins.

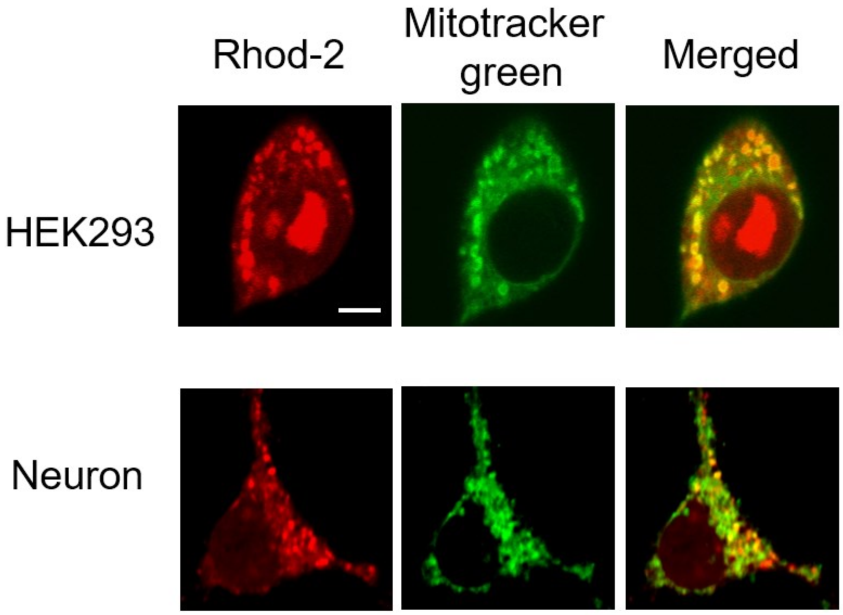


**Figure. S4**. HEK293 cells (upper panel) or primary cortical neurons (bottom panel) were stained with Rhod-2 (2.5uM) and mitochondrial green (100nM) for 20min at room temperature, washed and further incubated for 30min at 37°C. The images of double labeling were collected by Zeiss LSM 510 Meta Confocal Microscope. Scale bar = 5um. The experiments were replicated 3 times.

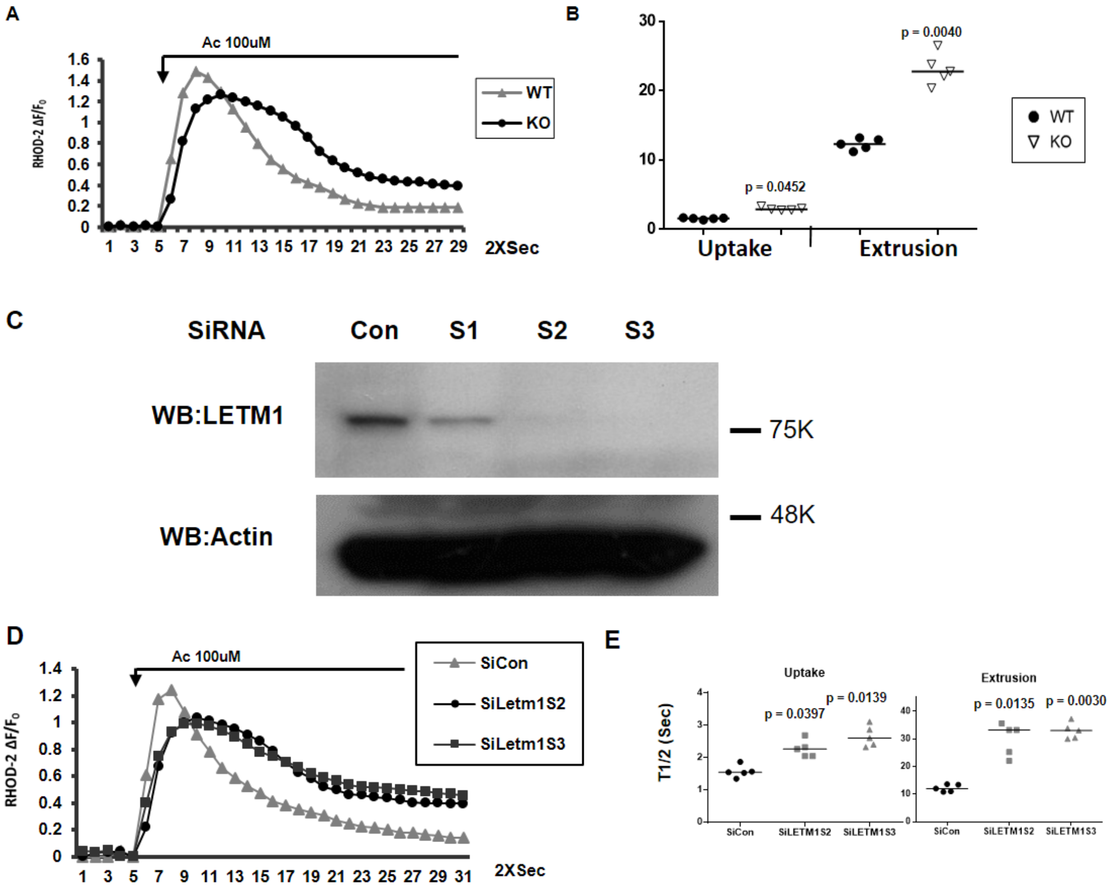


**Figure. S5**. (A) Representative fluorescence traces of mitochondrial $Ca^{2+}$ imaging in neurons from PINK1 WT and KO stimulated by Acetylcholine (Ac). (B) Quantification of the rate of calcium transport in (A) measured by $T_{1/2}$. n = 5. (C) SiRNA control (SiCon), SiRNA to LETM1–S1-3 were transfected in primary cortical neurons twice on DIV1 and DIV3 respectively. Neurons were collected at DIV6 and subjected to WB to validate the effect of SiRNA to LETM1. The experiment was replicated 3 times. (D) Representative fluorescence traces of mitochondrial $Ca^{2+}$ imaging in neurons transfected with control SiRNA (SiCon) or two sets of SiRNA to LETM1 (SiLETM1-S2 and S3) stimulated by Ac. (E) Quantification of the rate of calcium transport in (D) measured by $T_{1/2}$. n = 5.

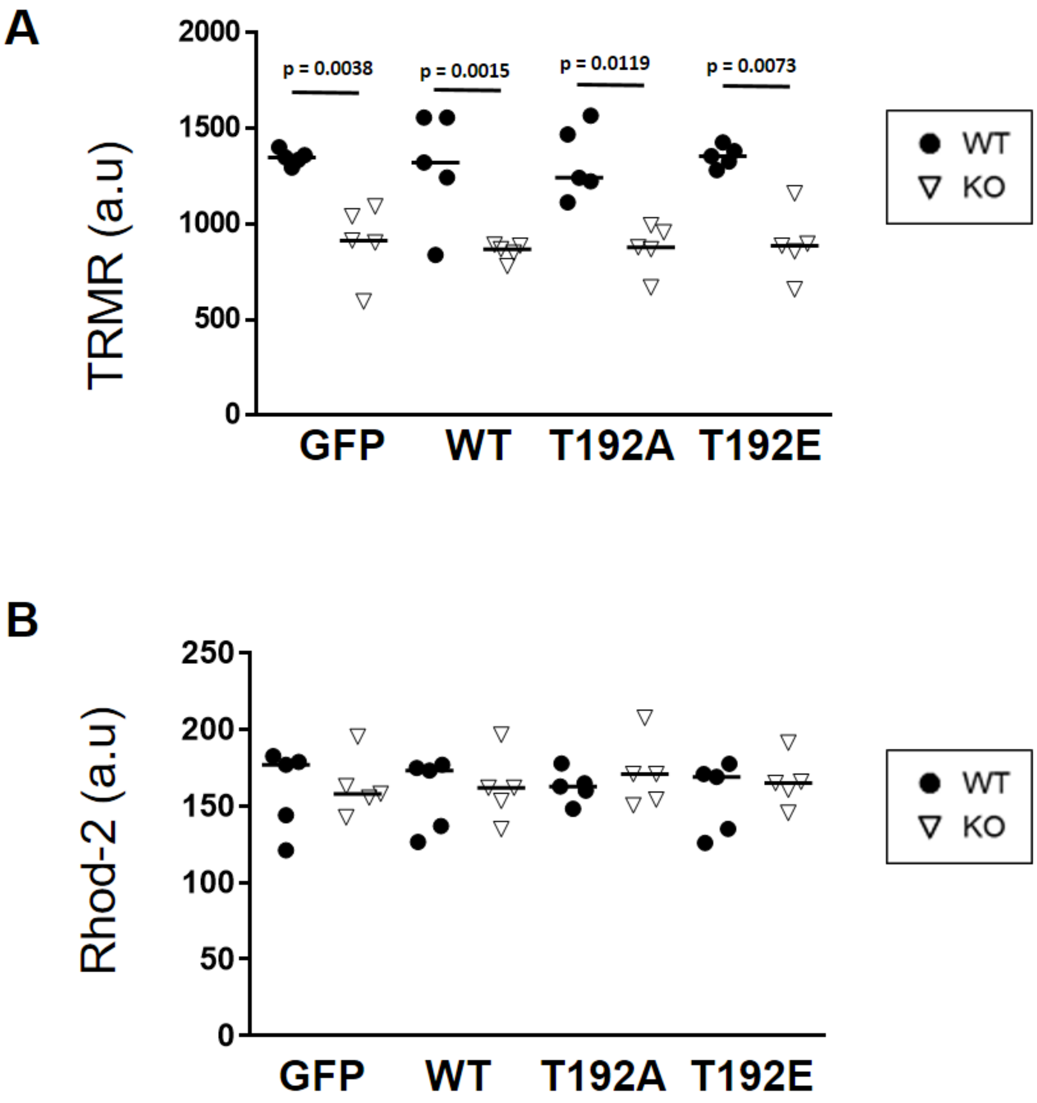


**Figure. S6.** (A) Quantification of the resting Δψm in PINK1 WT and KO neurons infected with adeno-viral GFP, LETM1 WT, T192A and T192E analyzed by TRMR(100ng/ml) staining for 20 minutes. n =5. (B) Quantification of the resting $[Ca^{2+}]_m$ in PINK1 WT and KO neurons infected with adeno-viral GFP, LETM1 WT, T192A and T192E analyzed by Rhod-2. n =5.

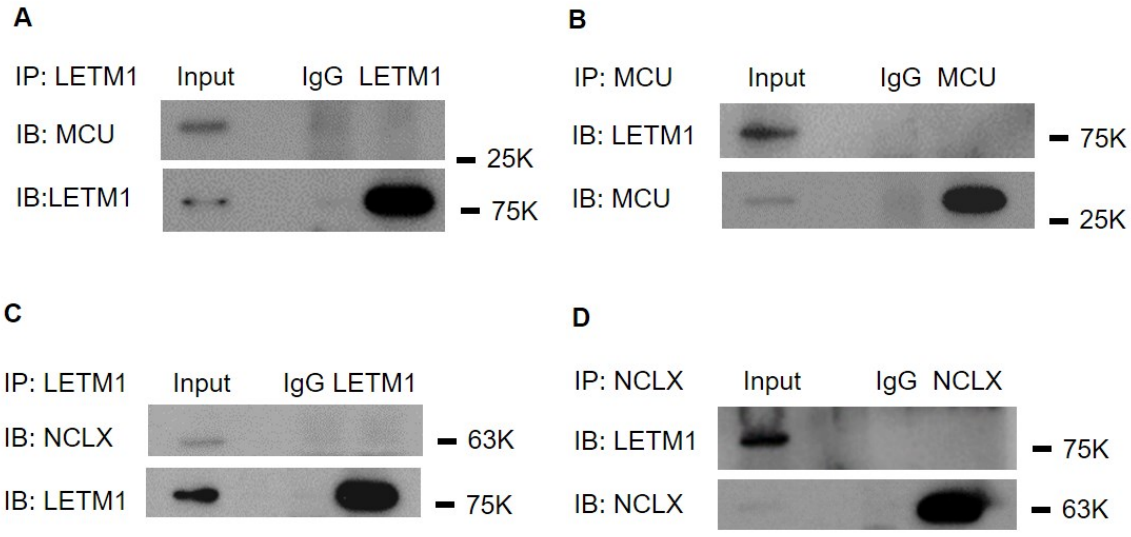


**Figure. S7.** LETM1 did not interacts with MCU and NCLX. (A) HEK cell lysate was IPed with control IgG or anti-LETM1 antibody. The precipitated proteins were probed with anti-MCU antibody and reprobed with anti-LETM1 by WB. (B) Inversely to (A), IP was performed with control IgG or anti-MCU, probed with anti-LETM1 and reprobed with anti-MCU antibody by WB. (C) HEK cell lysate was IPed with control IgG or anti-LETM1 antibody. The precipitated proteins were probed with anti-NCLX antibody and reprobed with anti-LETM1 by WB. (D) Inversely to (C), IP was performed with control IgG or anti-NCLX, probed with anti-LETM1 and reprobed with anti-NCLX antibody by WB. All above experiment was replicated 3 times.

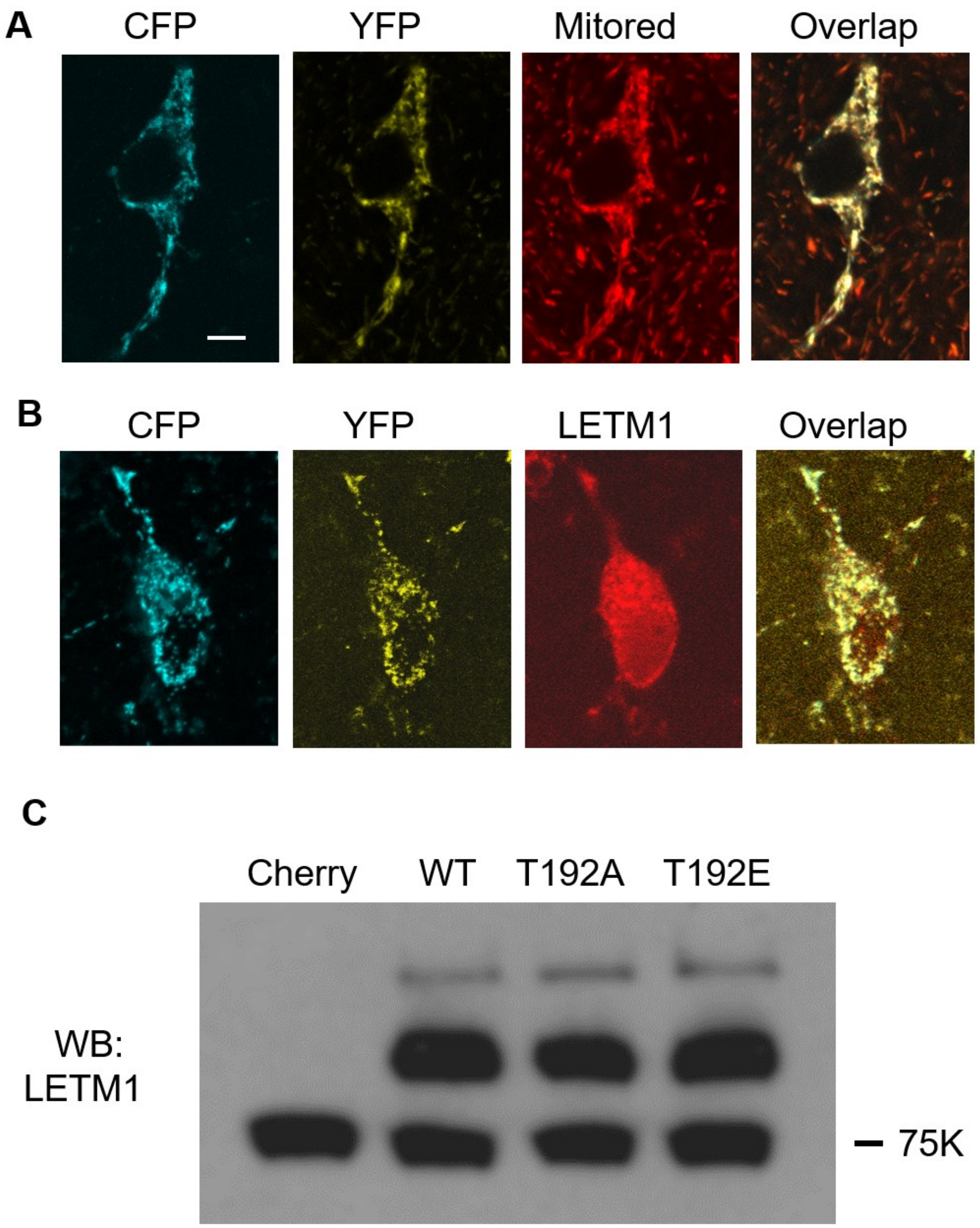


**Figure. S8.** (A) Neurons were infected with lenti-4XmTG-TN-XXL for 5days and stained with 100nM MitoTracker® Red CMXRos (Invitrogen, Cat: M7512) for 20 min. (B) Neurons were co-infected with lenti-4XmTG-TN-XXL and lenti-LETM1WT-3XFLAG-Cherry for 5 days. The images in A and B were collected by Zeiss LSM 510 Meta Confocal Microscope by CFP channel, YFP channel and Texas Red channel respectively. Scale bar = 5um. (C) Neurons were infected with lenti-mito-Cherry, LETM1-3XFLAG-Cherry WT, T192A and T192E for 5days. Cell lysates were subjected to Western blotting with anti-LETM1 antibody. The upper band showed LETM-3XFLAG variants and the lower band showed endogenous LETM. All above experiments were replicated 3 times.

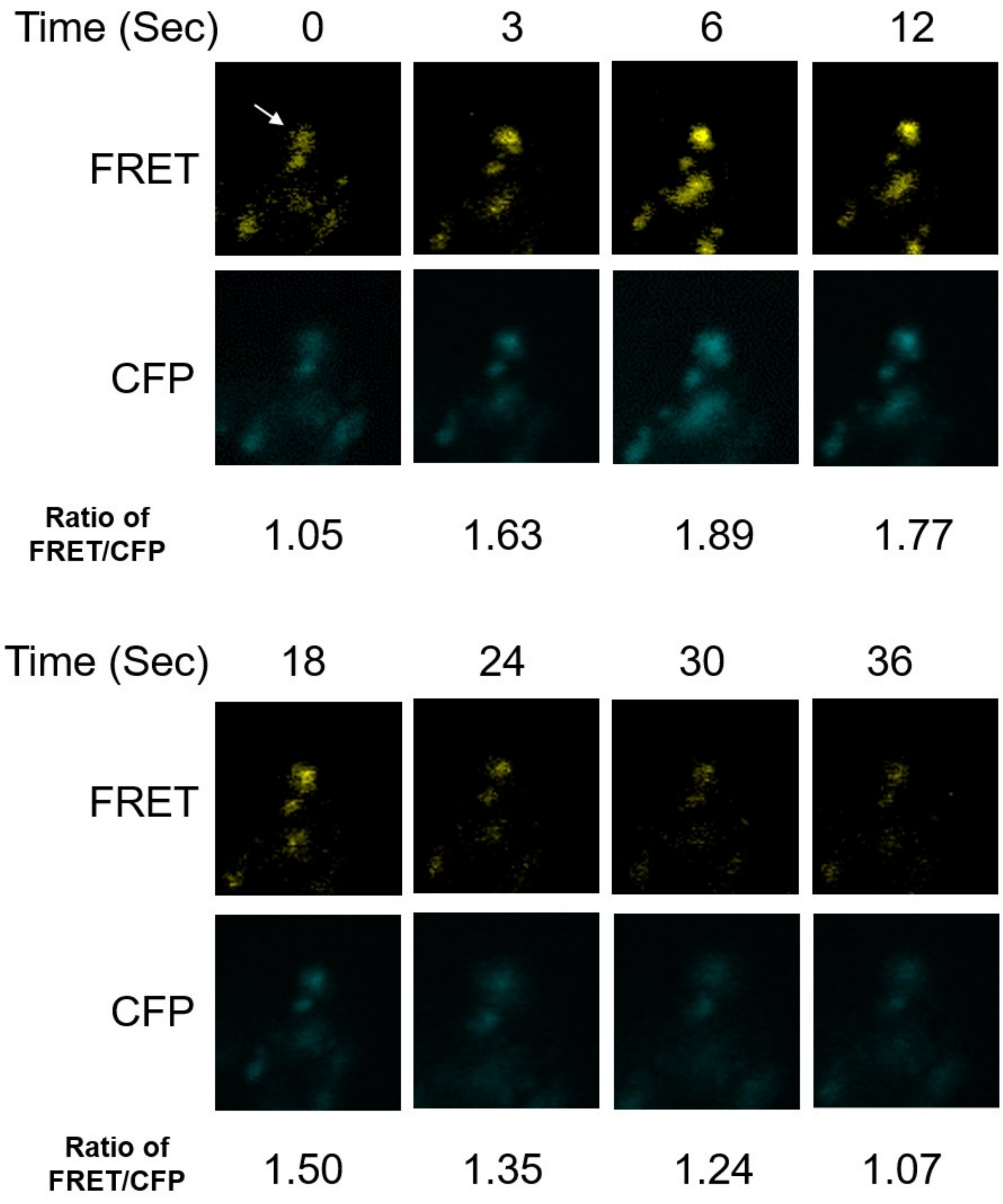


**Figure. S9.** Representative time lapse images in individual lenti-4mTG-TN-XXL infected neuron from PINK1-WT (corresponding to Fig. 4E) stimulated by 100uM Ac. The first image is the time point of Ac addition. The values of ratio of FRET/CFP in arrowed mitochondria are presented.

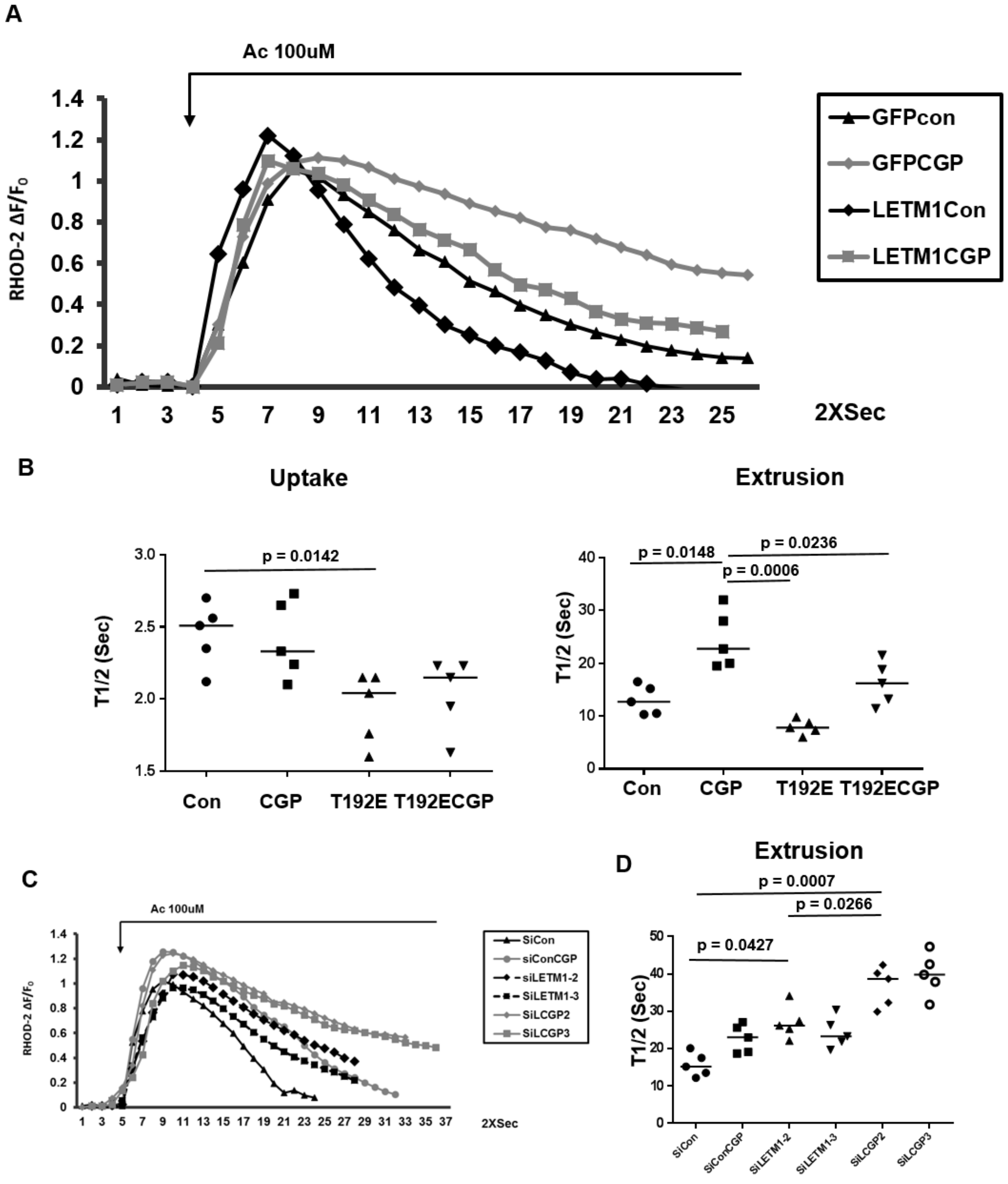


**Figure. S10.** LETM1 and NCLX regulate mitochondrial calcium extrusion independently. (A) Representative fluorescence traces of mitochondrial Ca2+ imaging in neurons from PINK1 WT infected with lenti GFP and LETM1 with DMSO (Con) or CGP37157(CGP) stimulated by Ac. (B) Quantification of the rate of calcium transport in (A) measured by T1/2. n = 5. (C) Representative fluorescence traces of mitochondrial Ca2+ imaging in neurons transfected with control SiRNA (SiCon) or two sets of SiRNA to LETM1 (SiLETM1-S2 and S3: SiL) with DMSO (Con) or CGP37157(CGP) stimulated by Ac. (D) Quantification of the rate of calcium extrusion in (D) measured by T1/2. n = 5

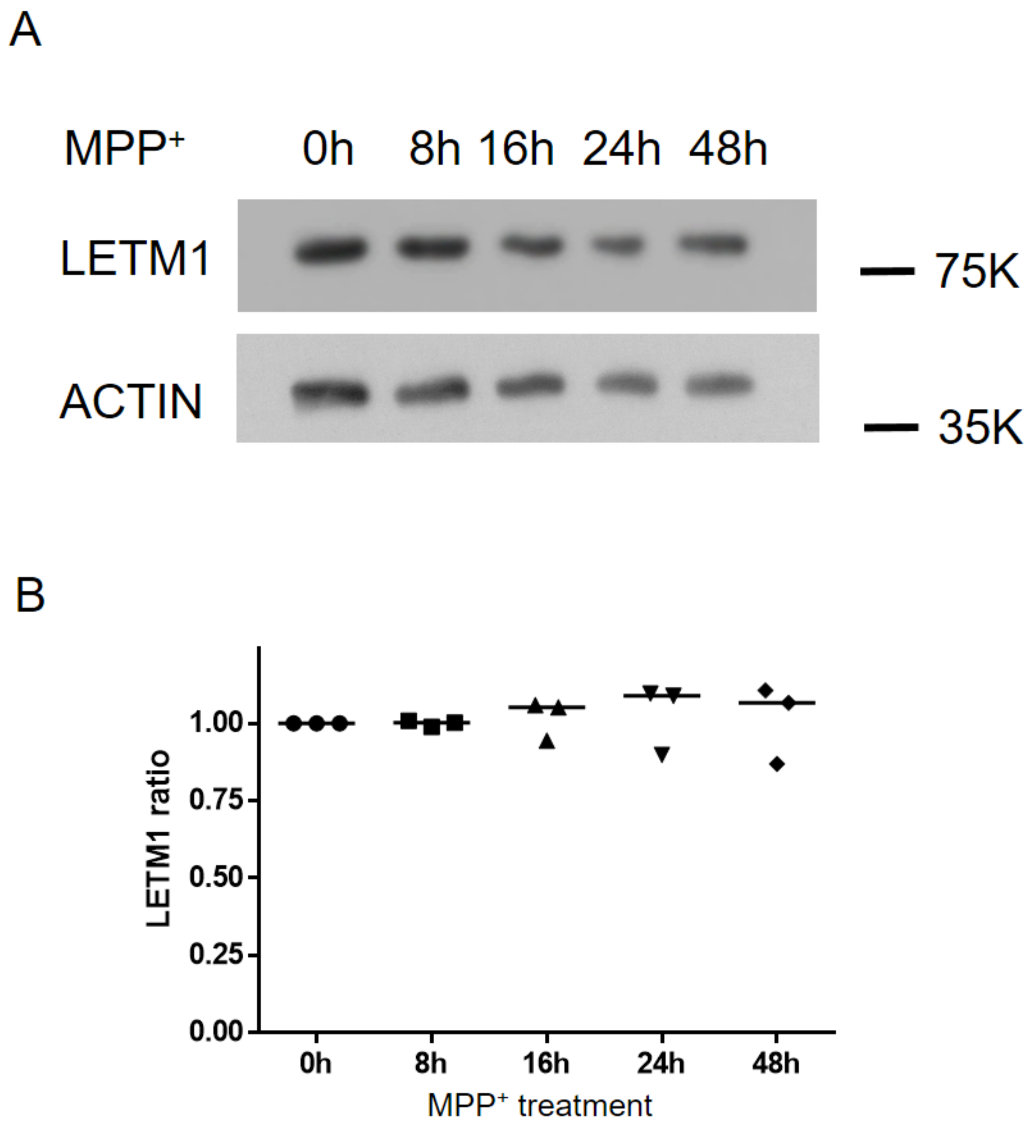


**Fig. S11**. The level of LETM1 protein in $MPP^+$ treated cortical neurons. (A). Cortical neurons were treated with MPP+ (20 μM) for 0h, 8h 16h, 24h and 24h and then processed to Western blot analyses. (B). Quantification of the densitometric values of LETM1 relative to Actin from western blots. n = 3.

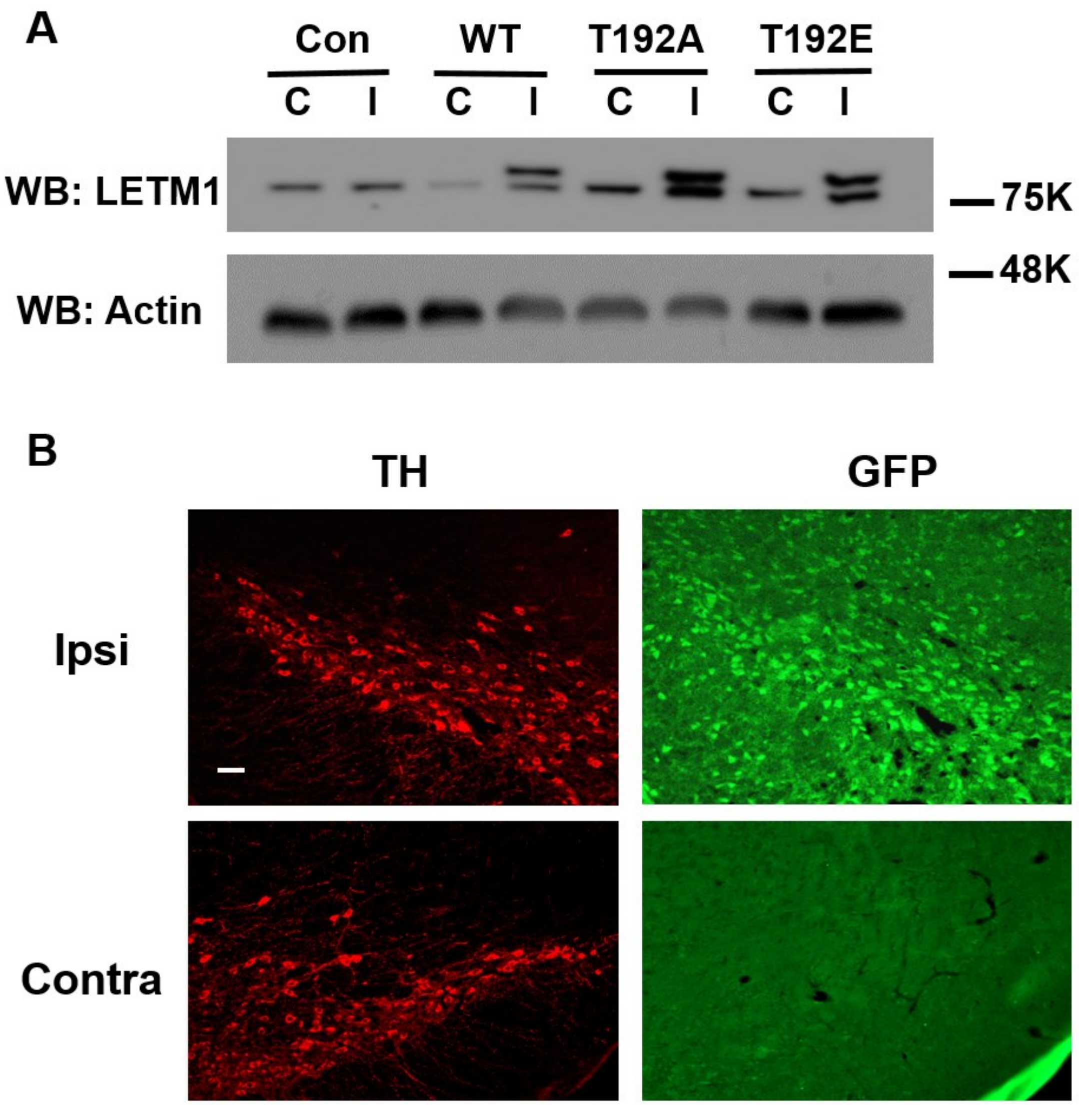


**Figure. S12.** Validation of viral expression in mice. (A) 7 days after viral injection, expression of Control GFP, LETM1-WT, T192A or T192E in the SNc extracts from PINK1 WT mice was confirmed by WB analyses using anti-LETM1 and actin antibody. The upper band showed FLAG-LETM1 variants and the lower band showed endogenous LETM1 (C, contralateral; I, ipsilateral). (B) Immunohistochemical localization of adenoviral expression of LETM1 WT in SNc of PINK1 WT mouse with viral injection post 7 days. Double-labeling immunofluorescence using mouse anti-GFP and rabbit anti-TH antibodies showed that adenoviral expression of GFP which was co-expressed with LETM1-WT (green) significantly localized to TH neurons (red) in the SNc area of the ipsilateral side. There is little GFP staining on the contralateral side. Scale bar: 50 μm. All above experiments were replicated 3 times.

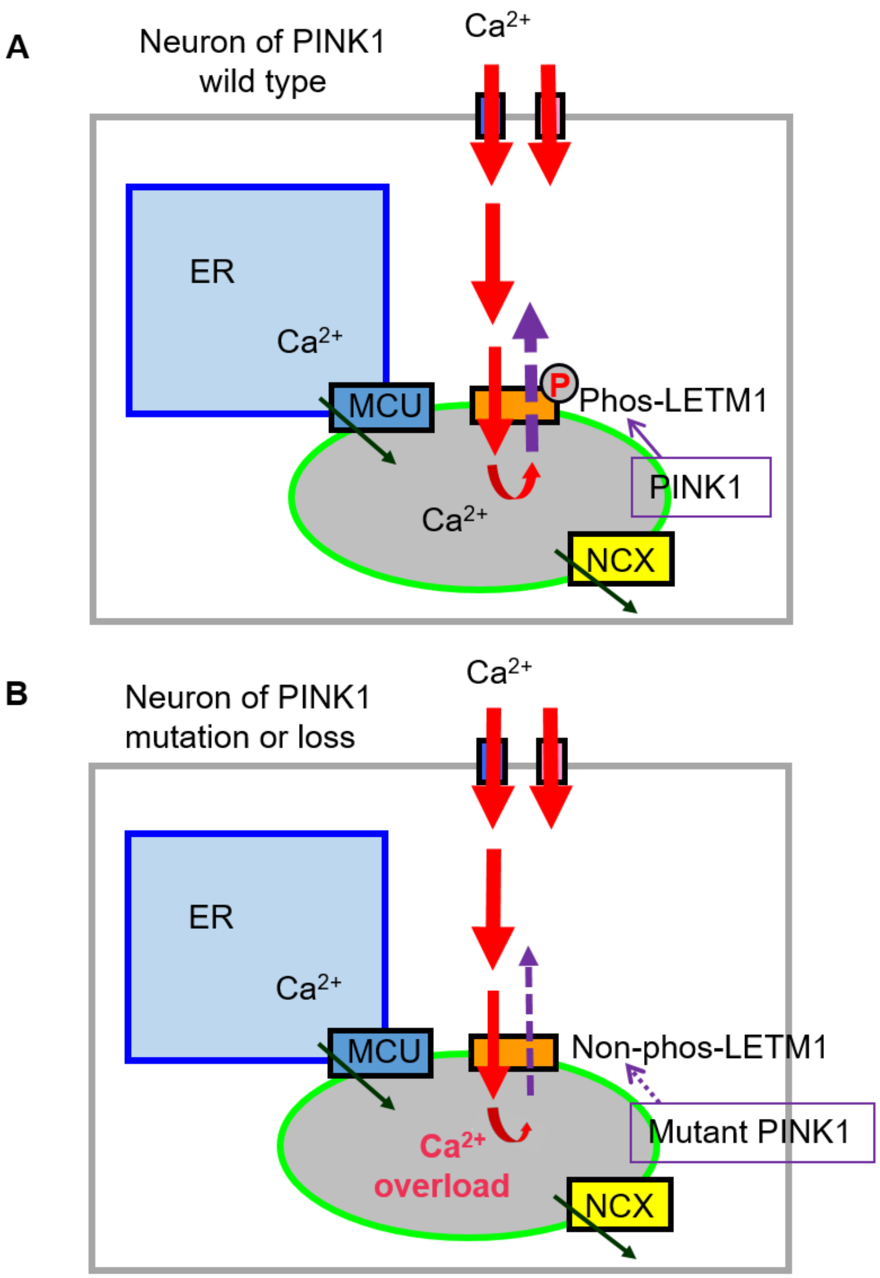


**Figure. S13.** Model for the role of PINK1-mediated phospho-LETM1 at Thr192 and mutant PINK1 in neuronal death. (A). Under pathological stress, extracellular $Ca^{2+}$ enters cytoplasm of neurons and is taken up by mitochondria leading to the elevation of $[Ca^{2+}]_m$. The PINK1-mediated phosphorylation of LETM1 at Thr192 enhances extrusion of mitochondrial $Ca^{2+}$ and prevents increase in $[Ca^{2+}]_m$. MCU: Mitochondrial Calcium Uniporter; NCX: $Na^+$- $Ca^{2+}$ exchanger; Arrows: $Ca^{2+}$ flows. (B). Mutant PINK1 reduces phosphorylation of LETM1 which compromises extrusion of mitochondrial $Ca^{2+}$, consequently lead to $[Ca^{2+}]_m$ overload and neuronal vulnerability.

# Uncropped Western blot images

Fig. 1A

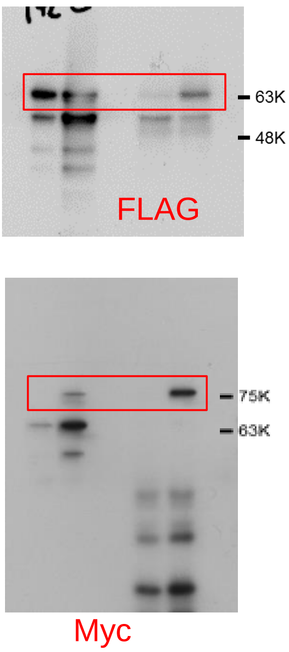


Fig. 1B

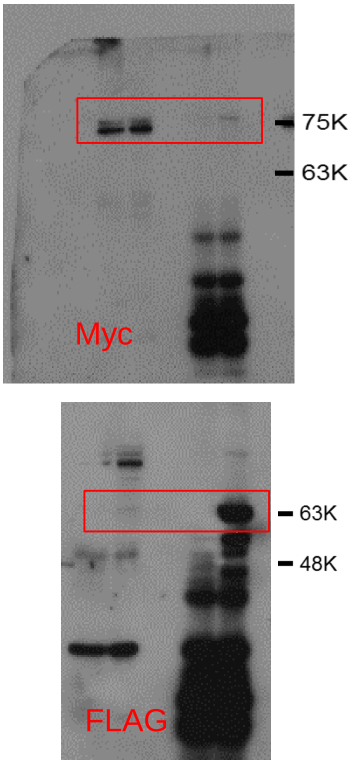


Fig. 1C

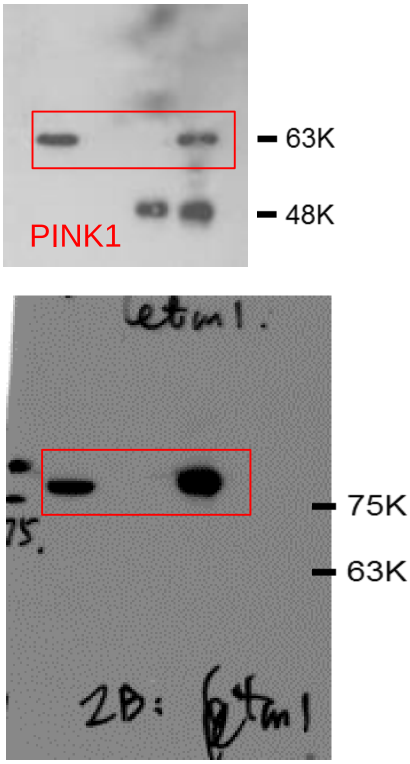


Fig. 1D

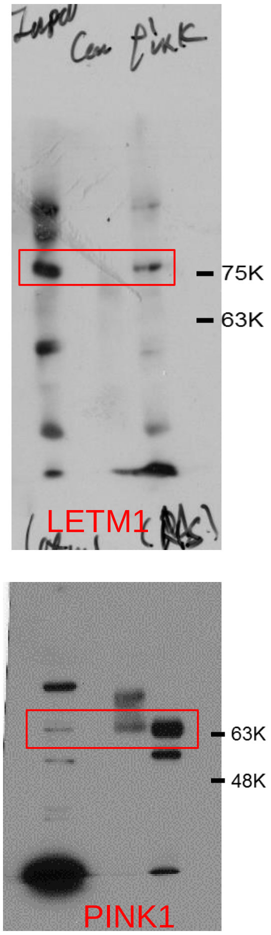


Fig. 1E

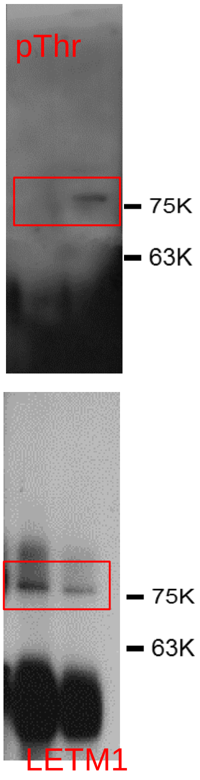


Fig. 1F

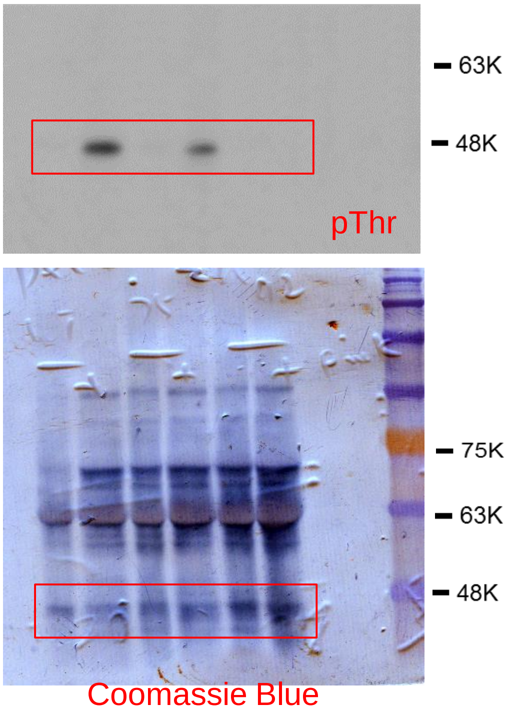


Fig. 1G

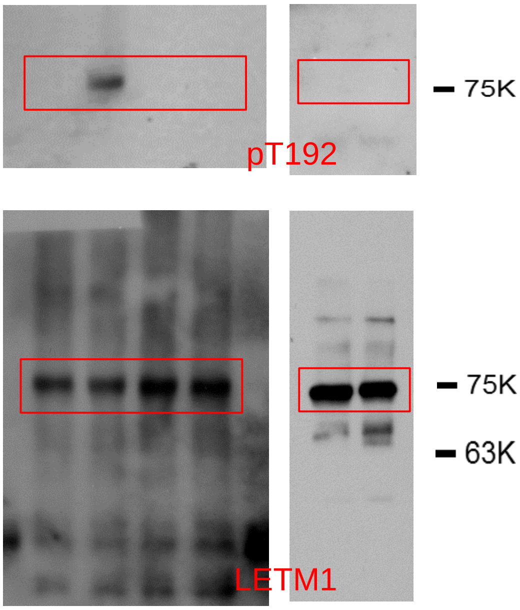

Fig.1H

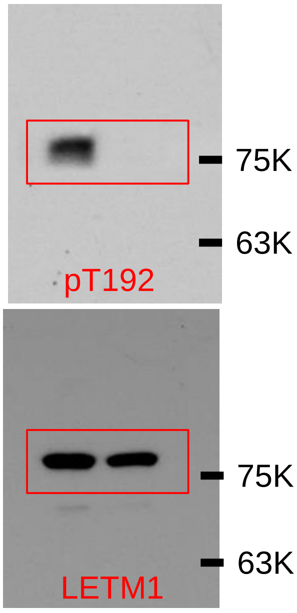


Fig. 2A

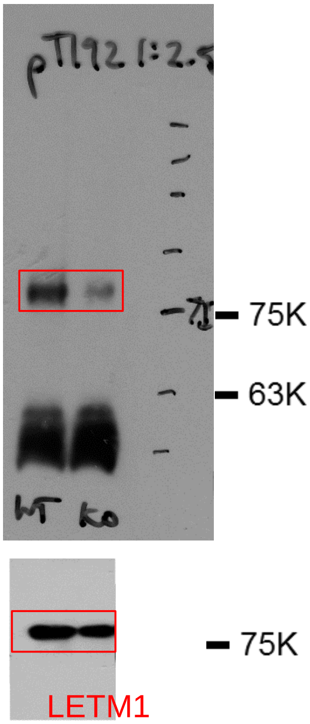


Fig. 2B

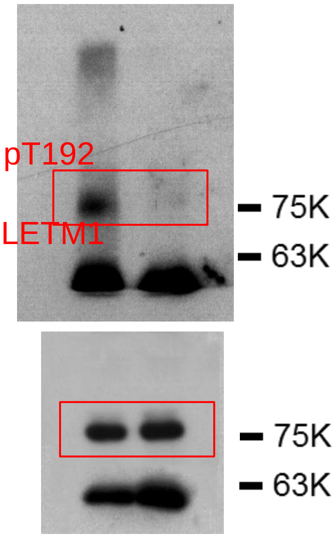


Fig.2C

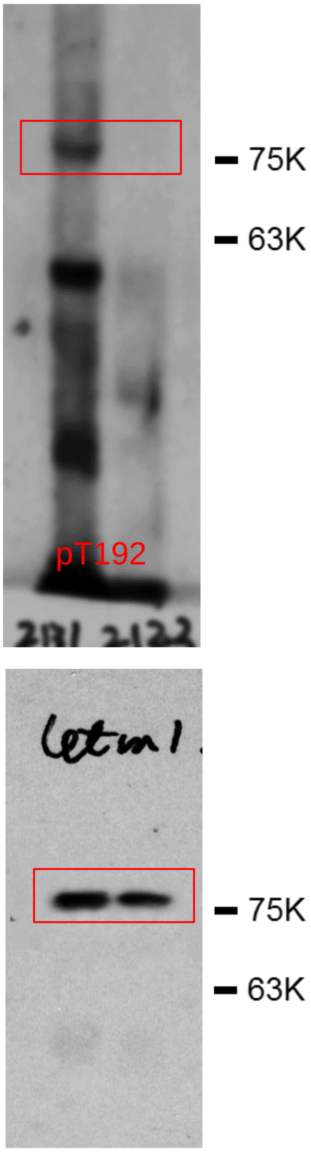


Fig.2D

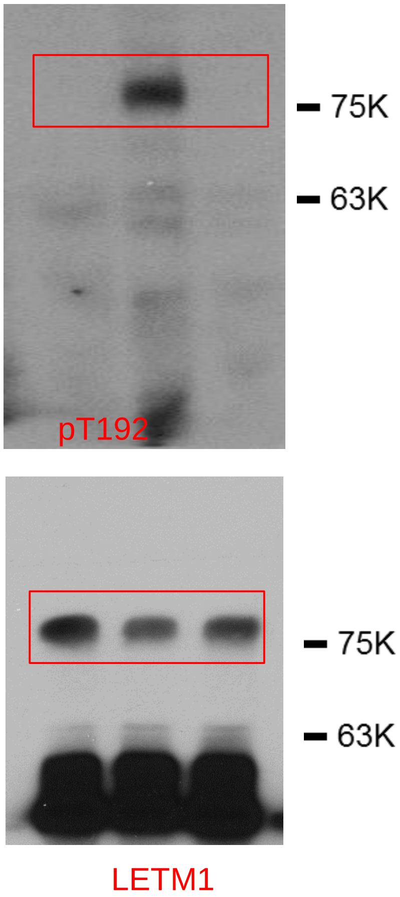


Fig.2E

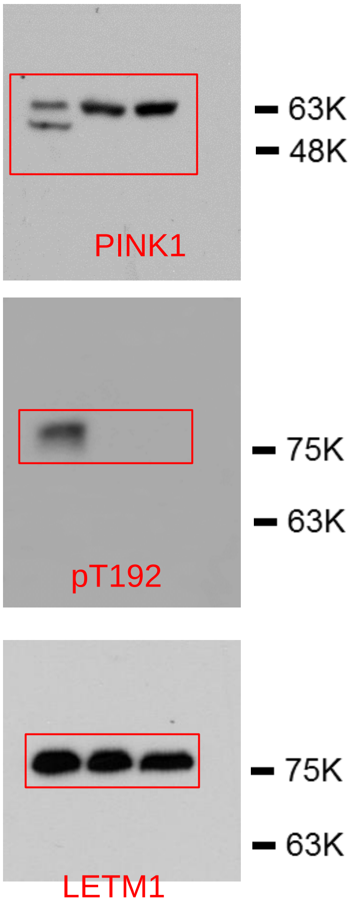


Fig. 3B

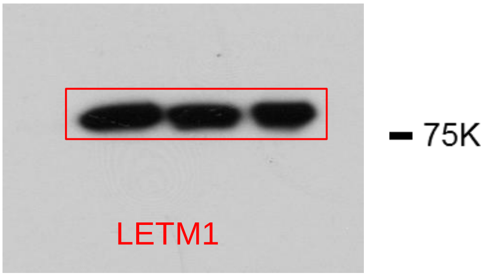

Fig. 3C

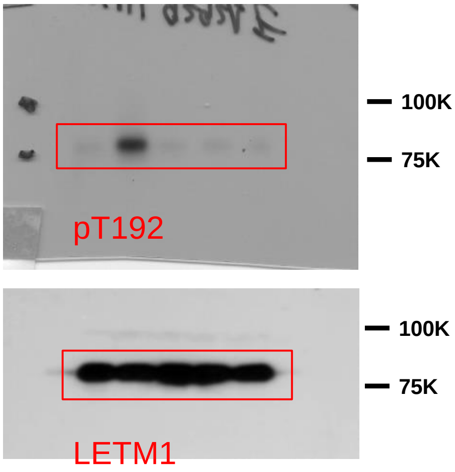


Fig. 3E

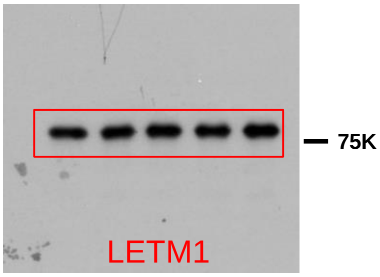


Fig. S1A

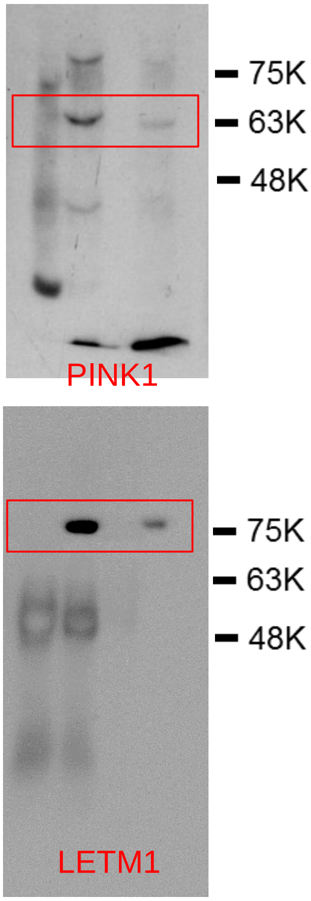


Fig. S1B

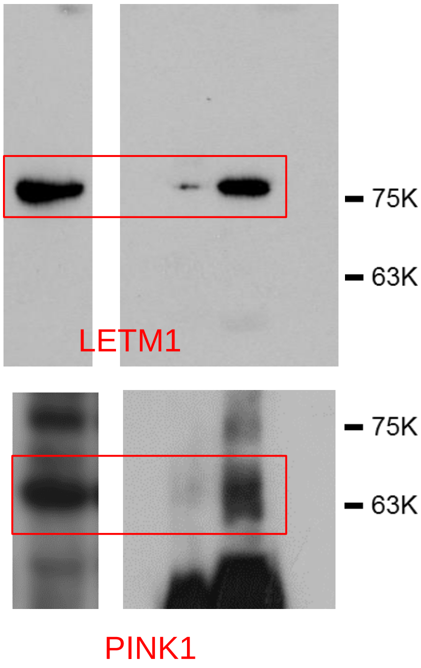


Fig. S1C

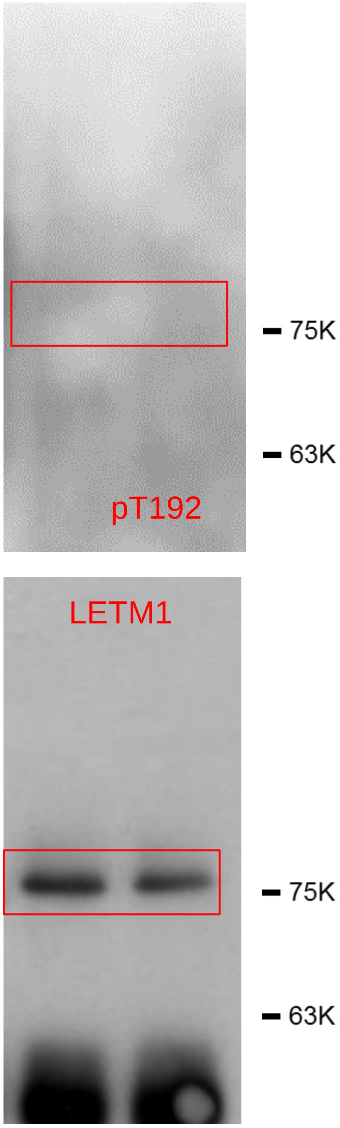


Fig. S1D

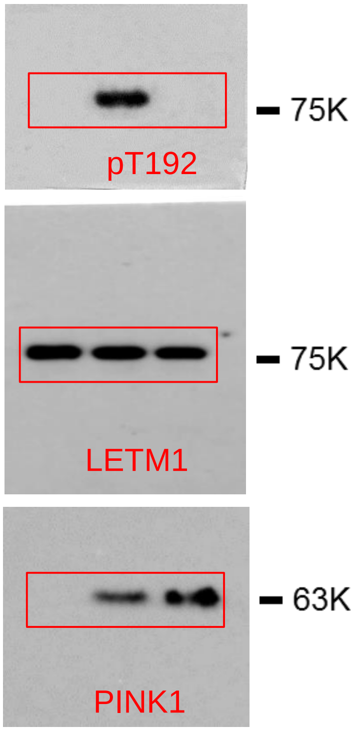

FigS3B

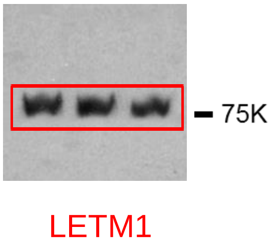


FigS3D

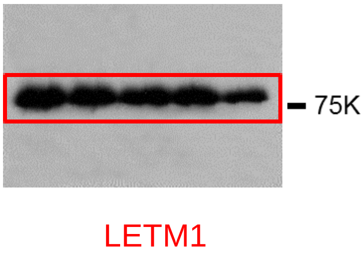


FigS5C

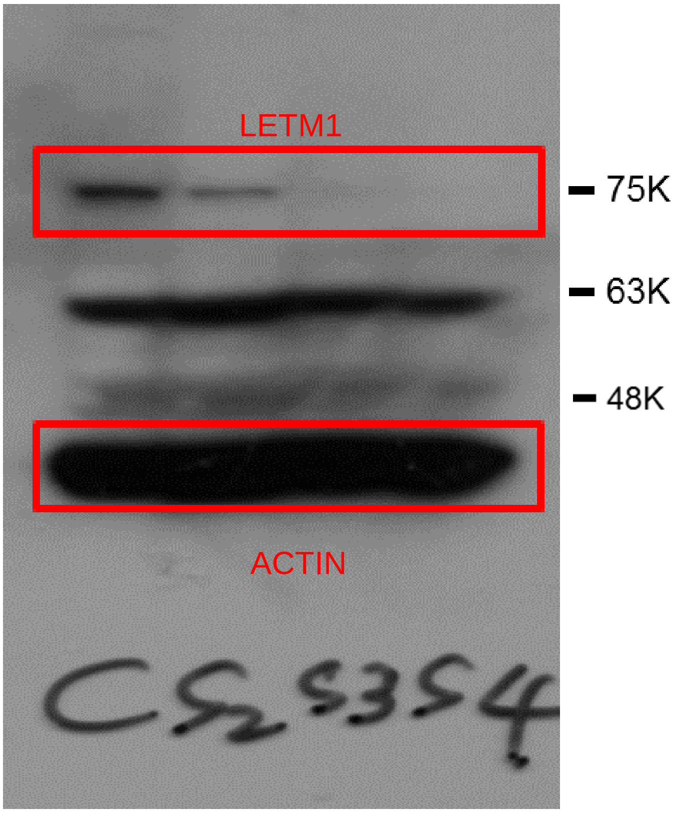


Fig. S7A

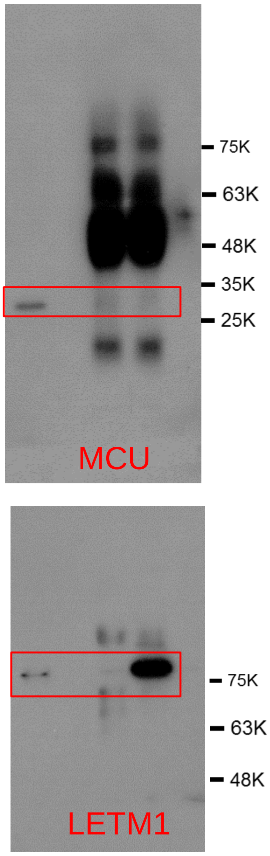


Fig. S7B

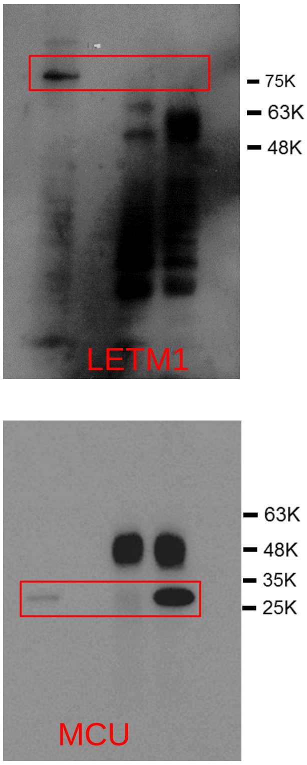


Fig. S7C

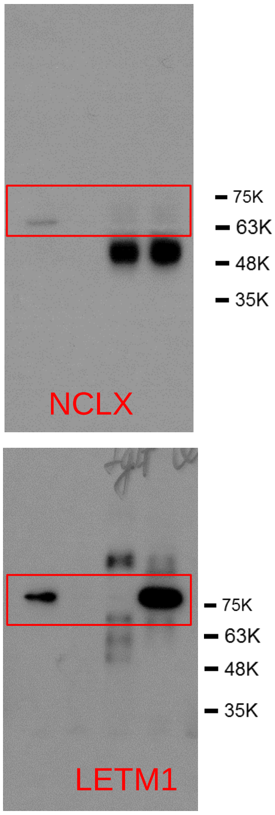


Fig. S7D

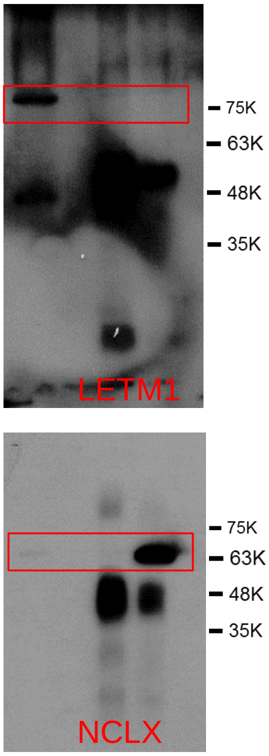

FigS8B

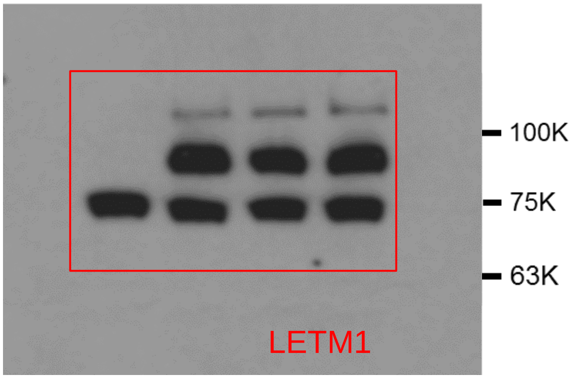


Fig. S11

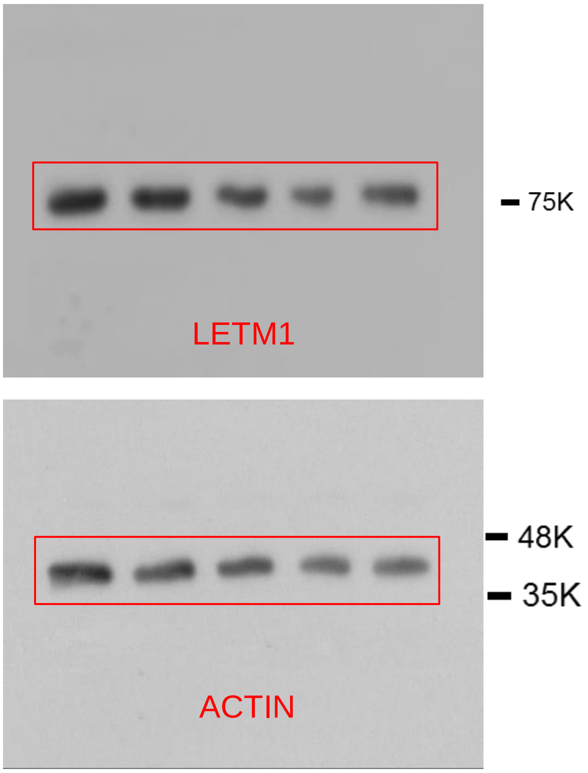


FigS12B

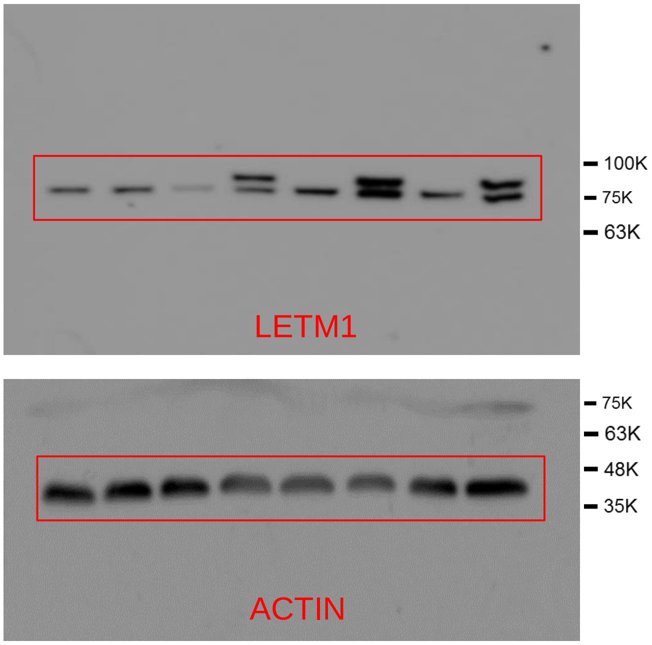

Supplement: Supplementary file 1 — Supplementary Information [file 41467_2017_1435_MOESM1_ESM.pdf]
